# NAE1-mediated neddylation coordinates ubiquitination regulation of meiotic recombination during spermatogenesis

**DOI:** 10.7150/thno.107843

**Published:** 2025-02-10

**Authors:** Yu Xi, Chenjia Gong, Zhe Zhang, Feiyin Zhu, Ying Zhang, Yanlin Tang, Liying Yan, Hui Jiang, Jie Qiao, Qiang Liu

**Affiliations:** 1 Center for Reproductive Medicine, Department of Obstetrics and Gynecology, Peking University Third Hospital, Beijing, China.; 2 State Key Laboratory of Female Fertility Promotion, Center for Reproductive Medicine, Department of Obstetrics and Gynecology, Peking University Third Hospital, Beijing, China.; 3 Department of Urology, Peking University Third Hospital, Beijing, China.; 4 National Clinical Research Center for Obstetrics and Gynecology (Peking University Third Hospital), Beijing, China.; 5 Key Laboratory of Assisted Reproduction (Peking University), Ministry of Education, Beijing, China.; 6 Beijing Key Laboratory of Reproductive Endocrinology and Assisted Reproductive Technology, Beijing, China.; 7 Peking-Tsinghua Center for Life Sciences, Peking University, Beijing, China.; 8 Department of Urology, Peking University First Hospital, Beijing, China.; 9 Institute of Urology, Peking University, Beijing, China.; 10 Department of Andrology, Peking University First Hospital, Beijing, China.

**Keywords:** meiosis, NAE1, neddylation, ubiquitination, homologous recombination, spermatogenesis

## Abstract

**Rationale:** Meiotic homologous recombination is a critical event in gametogenesis, which is tightly regulated to ensure the generation of crossovers on homologous chromosomes. This process is crucial for ensuring the accurate segregation of genetic material and maintaining genetic diversity within species, ultimately contributing to reproductive success. Nevertheless, comprehensive mechanisms of post-translational modification (PTM) regulating homologous recombination during meiosis require further investigation. The aim of this study is to investigate the regulatory mechanisms and physiological functions of NAE1-mediated neddylation during meiosis of mammalian spermatogenesis and its consequential role in infertility.

**Methods:** The dynamic localization of NAE1 at various sub-stages during spermatogenesis was determined using immunofluorescence staining and seminiferous tubule staging. We explore the role of NAE1-mediated neddylation by utilizing germ cell-specific *Nae1-*knockout mice. The impact on homologous synapsis and recombination during the meiosis prophase I were verified through chromosome spread fluorescence staining. We used 10 × Genomics single cell transcriptomics and ubiquitinomics to analysis the causes of spermatogenesis arrest and spermatogenic apoptosis.

**Results:** NAE1 exhibited high nuclear expression within spermatocytes from the pachytene stage onwards. *Nae1*-SKO male mice showed a late-pachytene arrest in spermatocytes, resulting in infertility. In NAE1-deficient spermatocytes, there is an increase in apoptosis. *Nae1* deletion led to double-strand break (DSB) repair failure with normal autosomes synapsis. From a mechanistic perspective, we verified excessive recombination intermediate stabilization and failed crossover formation, which ultimately resulted in impaired meiotic recombination. Further analysis showed that ubiquitination regulation coordinated with NAE1-mediated neddylation was implicated in meiotic recombination.

**Conclusion:** NAE1-mediated neddylation regulates ubiquitination during meiosis and is involved in the stabilization of recombination proteins related to crossover differentiation. We provide cytological evidence for the neddylation-ubiquitination system (NUS) in mammalian meiotic recombination during spermatogenesis.

## Introduction

Meiosis is a critical event in gametogenesis that is essential for reproductive success. Prophase I of meiosis is generally divided into four substages based on the appearance of chromosomes: leptotene, zygotene, pachytene, and diplotene 1. Germ cells undergo pairing and recombination of homologous chromosomes during this process, accompanied by significant shifts in gene expression 2 and dynamic alterations in chromatin configuration 3. Impaired meiosis may result in aneuploidy and even infertility 4.

Homologue pairing and segregation success rely on homologous recombination, which generates genetic variability 5. Initially, programmed double-strand breaks (DSBs) were induced by the topoisomerase VI complex proteins SPO11 and TOP6BL, which catalyze the initiation of meiotic recombination 6, 7. Next, 3' single-strand DNA tails generated by end resection initiate a homology search and invade the homologous chromosome with the aid of RAD51 and DMC1, thus forming displacement loops (D-loops). The capturing of the 5' end by the D-loop and subsequent connection with the 3' end generate a double Holliday junction (dHJ) intermediate 8. Finally, homologous recombination is completed with different dHJ dissociation modes, forming crossovers and non-crossovers 9. The ratio of DSBs to crossover numbers indicated that the majority (approximately 90%) of DNA strand invasion intermediates were resolved with non-crossovers. However, at least one crossover is typically observed in each pair of homologues 10. Different crossover and non-crossover pathways exhibit marked differentiation in the stabilization of strand-invasion intermediates and genetic requirements. A dozen pro-crossover factors (some of which remain unknown) participate in the facilitation and/or regulation of crossover differentiation and maturation, including the MSH4/MSH5 (MutSγ complex), which plays a key regulatory role. This clamp-like heterodimer stabilizes DNA strand invasions to bind branched recombination intermediates 11. Approximately 90-95% of crossovers are generated by the class I pathway, which is reliant on the direct stimulation of DNA cleavage by the MutSγ complex recruiting MLH1/MLH3 (MutLγ) 12.

Post-translational modifications (PTMs) were deemed to play necessary roles in the progression of meiosis prophase I, with examples such as ubiquitination and SUMOylation attracting significant attention for their critical regulatory functions 13, 14. The number of studies focusing on homologous recombination during spermatogenesis has rapidly increased over the decades; however, the comprehensive mechanisms of PTM regulation remain uncertain. Neural precursor cell-expressed developmentally down-regulated 8 (NEDD8) exhibits a high degree of homology with ubiquitin 15. Neddylation, which has gradually gained research attention over time, is the process of covalently and reversibly tagging NEDD8 to the lysine residue of a substrate protein, thereby regulating its stability, activity, or function 16. Similar to the process of ubiquitination, NEDD8 conjugation to target proteins is mediated through an enzymatic cascade involving three distinct enzymes. This cascade commences with the NEDD8-activating enzyme E1, and then transfers to the NEDD8-conjugating enzyme E2, and ultimately its conjugation to specific substrates by E3 ligase 17. Among the known neddylation substrates, the Cullin family proteins are considered the primary physiological targets, and the conjugation of NEDD8 to the C-terminal lysine residue of Cullins is crucial for the activation of Cullin-RING ligases (CRLs) 18.

So far, the only identified NEDD8-activating enzyme E1 is a heterodimer that comprises the regulatory subunit NAE1/APP-BP1 and the catalytic subunit UBA3 19. The NAE1 homologue, AXR1, is a regulator of meiosis in *Arabidopsis* 20. However, the physiological functions of NAE1 and its regulatory mechanism in mammalian meiosis have not been elucidated. Herein, we report that NAE1 deficiency leads to infertility in male and female mice. The progression of *Nae1^fl/-^; Stra8-Cre* spermatocytes are arrested in late-pachytene accompanied by an apoptotic increase. Furthermore, the loss of NAE1 interferes with the neddylation and ubiquitination in spermatocytes, which in turn leads to excessive stabilization of meiotic recombination intermediates, and inability to repair DSBs completely, failure of crossover formation and ultimately recombination process obstruction. Our results revealed an indispensable crosstalk between NAE1-activated neddylation and ubiquitination during meiotic recombination.

## Materials and methods

### Ethics statement

All the experiments on mice followed the guidelines of the Institutional Animal Welfare and Ethics Committee policies of Peking University with the approval number LA2021579.

### Mice

Mice lacking *Nae1* in male germ cells were generated by crossing with *Stra8-Cre* mice. The founder mice were backcrossed onto the C57BL/6 background. Newborn mice were genotyped using polymerase chain reaction (PCR) and subsequent DNA sequencing analysis of samples extracted from their tails. The mice were housed under a controlled 12-hour light/12-hour dark cycle at an ambient temperature of 23 ± 3 ℃ in the specific pathogen-free facility at Peking University Health Science Center. They were provided with ad libitum access to food and water throughout the study.

### Histological analysis and immunofluorescence staining

The mice were euthanized via cervical dislocation, and their testes, cauda epididymis, and ovaries were harvested. These organs were then fixed in Bouin's solution for Hematoxylin and Eosin (HE) and Periodic Acid-Schiff (PAS) staining or in 4% paraformaldehyde for TUNEL and immunofluorescence staining, both at 4 ℃ overnight. The samples underwent a series of ethanol dehydration steps before being embedded in paraffin and sectioned into 5 µm-thick slices. The paraffin sections were stained with hematoxylin and eosin (cat# BA4097 and cat# BA4098, BaSO, Zhuhai, China), and imaged using digital panoramic scanner (WISLEAP, Zhiyue Medical Technology Co., Ltd; Jiangsu, China). Sections were first deparaffinized and rehydrated, followed by staining with the PAS reagent (R20526, Yuanye Bio-Technology, Shanghai, China) and counterstaining with hematoxylin. Afterward, the tissue sections were dehydrated, cleared, and sealed with neutral resin. For the detection of apoptosis in testicular sections, a terminal deoxynucleotidyl transferase dUTP nick end labeling staining kit (#C1091, Beyotime Biotechnology Inc., Haimen, China) was employed.

In preparation for immunofluorescence analysis, the paraffin sections were dewaxed and rehydrated before being subjected to antigen retrieval by heating at 95 ℃ in EDTA antigen repair buffer (ZLI-9071, ZSGB-BIO; Beijing, China) for 30 min, followed by gradual cooling to room temperature. The sections were then rinsed in phosphate-buffered saline (PBS), blocked with 10% donkey serum in 0.01 M PBS for 1 hour at room temperature, and subsequently incubated with the primary antibodies at 4 ℃ overnight. After three PBS washes, the sections were treated with the appropriate secondary antibodies and 4,6-diamidino-2-phenylindole (DAPI) for 1 hour at 25 ℃. The samples were then washed three times in PBS, mounted, and examined using a confocal microscope (LSM880, Carl Zeiss AG, Oberkochen, Germany). Detailed antibodies used in this study are listed in Table S1.

### Spermatocyte nuclear surface spreading and immunofluorescence staining

Mouse testes were carefully dissected to remove the tunica albuginea, and the seminiferous tubules were meticulously isolated. These tubules were then incubated in a hypotonic buffer solution composed of 30 mM Tris, 50 mM sucrose, 17 mM trisodium citrate dihydrate, 5 mM EDTA, 0.5 mM DTT, and 0.5 mM phenylmethylsulfonyl fluoride, adjusted to pH 8.2, for 30-40 minutes at room temperature. Following incubation, the fragmented testicular tubules were transferred to a 0.1 M sucrose buffer at pH 8.2 to facilitate the dispersion of cells into a single-cell suspension. This suspension was gently spread onto microscope slides precoated with 1% paraformaldehyde (PFA) fixative buffer containing 0.15% Triton X-100 at pH 9.2. Specifically, 10 μL of the cell suspension was carefully layered with 45 μL of the same 1% PFA fixative buffer containing 0.15% Triton X-100 from the edge of clean, adhesive microscope slides (Citotest Scientific Co., Ltd., Nanjing, China). The slides were then fixed for 1 hour in a humidified chamber at room temperature before being air-dried and washed three times with a 0.2% solution of PhotoFlo (Kodak, Rochester, NY) to prepare for blocking. For immunostaining, the slides were first blocked with 10% donkey serum in Tris Buffered Saline containing 0.1% Tween 20 (TBST) for 1 hour at room temperature. They were then incubated with the primary antibody overnight at 4 ℃ overnight. After three washes with TBST, the slides were incubated with the corresponding secondary antibodies for 1 hour at 37 ℃. Following another three washes in TBST, the slides were mounted, and the images were captured using a confocal microscope. Detailed antibodies used in this study are listed in Table S1.

### Western blotting

Mouse testes and other tissues were lysed using RIPA buffer (P0013B, Beyotime Biotechnology, Inc., China). The homogenates were centrifuged at 13,500 g for 15 min at 4 ℃. The resulting homogenates were centrifuged at 13,500 g for 15 minutes at 4 ℃ to separate the supernatants. Protein concentrations in the supernatants were determined using a bicinchoninic acid (BCA) assay kit (Pierce/Thermo Fisher Scientific, Waltham, MA). Equal amounts of protein samples were then resolved by sodium dodecyl sulfate-polyacrylamide gel electrophoresis (SDS-PAGE) and transferred onto a polyvinylidene difluoride (PVDF) membrane (Bio-Rad, Hercules, CA). The membranes were blocked with 5% non-fat milk in Tris-buffered saline containing 0.5% Tween-20 buffer (B1009, Applygen Technologies Inc. Beijing, China) for 30 min at room temperature. After an overnight incubation with the primary antibodies at 4 ℃, the membranes were washed three times in TBST and then incubated with horseradish peroxidase (HRP)-conjugated secondary antibodies in 5% non-fat milk for 1 hour at room temperature, followed by another three washes in TBST. For signal detection, an enhanced chemiluminescence detection kit (32106, Thermo Fisher Scientific) was applied to the membranes, and the signals were captured using the Tanon 5200 chemiluminescence detection system (Tanon, Shanghai, China). Detailed first and second antibodies used in this study are listed in Table S1.

### Fluorescence-activated cell sorting

Various germ cells were isolated from the testes of male mice using fluorescence-activated cell sorting (FACS). Briefly, after removing the tunica albuginea, the testes were immersed in 5 ml of DPBS and treated with 120 U/ml collagenase I (17100017, Thermo Fisher Scientific) for 10 minutes at 34℃. Subsequently, the seminiferous tubules were further digested for 8 minutes at the same temperature with 5 ml of 0.25% trypsin (25200072, Gibco) and 0.1 ml of 5 mg/ml DNase I (DN25, Sigma-Aldrich). The digestion was terminated by adding 0.5 ml of fetal bovine serum (FBS). The cell suspension was filtered through a 70 µm filter and centrifuged at 500 g for 5 minutes at 4℃. The pellet was resuspended in DMEM containing 5µl of DNase I. To prevent Hoechst-induced cell death due to excessively high localized concentrations, staining was performed in two steps. First, Hoechst was added at a 1:2000 dilution based on the final diluted volume. The centrifuge tube was placed horizontally in the hybridization incubator and incubated at the lowest rotation speed for 10 minutes. After incubation, 4 times the volume of Hoechst staining solution was added, and the tube was returned to the hybridization incubator for low-speed rotation incubation for 20 minutes. After staining was complete, centrifugation was performed at 500 g for 5 minutes at 4℃, the supernatant was discarded, and 500µl of a mixture (DMEM:FBS:DNase I=500:10:1) was added to resuspend the cells. Cells were stained with propidium iodide (25535-16-4, Sigma-Aldrich) at room temperature and then filtered through a 40 µm filter. Cell populations were sorted using a flow cytometer (BD Biosciences, FACS Aria II, USA) based on the fluorescence intensity of Hoechst 33342/propidium iodide staining. Hoechst was excited by a 355 nm UV laser.

### Staging of seminiferous tubule on testis paraffin sections

The staging of mouse seminiferous tubule sections with immunofluorescence 21 and PAS staining 22, 23 was determined based on previous reports.

### Quantification of recombination foci number and the fluorescence intensity

The variety and number of recombination foci in spread spermatocytes were examined using the ZEISS ZEN 3.4 (blue edition) software, with manual counting of the foci. The fluorescence intensity of HEI10 foci and FK2 was measured utilizing ImageJ (National Institutes of Health, USA). To determine the mean background fluorescence intensity, areas adjacent to the immunofluorescence foci (HEI10) or along the autosome axis (FK2) were selected for analysis. The fluorescence intensity of the XY body area was excluded from the total fluorescence intensity calculation for FK2.

### Single cell RNA-seq

In each single-cell RNA sequencing experiment, frozen testis tissue was rapidly thawed and processed. The samples were first washed twice with PBS, then scraped using razor blades. They were treated with a mixture of 1 mg/ml collagenase type IV and 1 mg/ml DNase I, followed by incubation with trypsin-EDTA and 1 mg/ml DNase I at 37 ℃ for 5 minutes. Subsequently, the resulting single cells were filtered through 70 μm strainers (Miltenyi Biotec, Bergisch Gladbach, Germany) and rinsed with Dulbecco's PBS (DPBS). The cells were then resuspended in DPBS supplemented with 0.4% bovine serum albumin (BSA, B2064, Sigma, St Louis, MO) at a concentration of 1,000 cells/ml, ready for single-cell sequencing. The experimental protocol adhered to the guidelines provided by 10 × Genomics for the Chromium Next GEM Single Cell 3' Reagent Kits v3.1. For the formation and encapsulation of Gel Bead in Emulsions (GEMs), cells were diluted to approximately 5,000 cells per lane and combined with the master mix on the Chromium Next GEM Chip G. After the post-GEM-RT cleanup, cDNA amplification was performed for 12 cycles. The resulting libraries were analyzed on an Illumina NovaSeq 6000 platform by Annoroad Gene Technology Co., Ltd (Beijing, China) with the following sequencing parameters: 28 cycles for Read 1, 10 cycles for i5 index, 10 cycles for i7 index, and 90 cycles for Read 2.

### Differentially expressed genes and functional enrichment analysis

We utilized DESeq2 (version 1.26.0) to ascertain the adjusted *P*-values and fold changes for the identification of differentially expressed genes (DEGs). Genes were classified as DEGs if they exhibited an adjusted *P*-value of less than 0.01 and a fold change greater than 1.5. For the functional annotation of these DEGs, we employed the DAVID Bioinformatics Resources 6.8 platform to perform Gene Ontology (GO) analyses. GO enrichment analysis was applied to both upregulated and downregulated proteins, with a focus on three ontological domains: Biological Process, Molecular Function, and Cellular Component. The reference gene set was constituted by all proteins detected within the dataset. The statistical significance of the GO term enrichment was assessed using hypergeometric tests, with P-values adjusted for multiple comparisons. The ggplot2 package was then applied to graphically represent the top ten most significantly enriched terms within each category.

### RNA extraction and quantitative polymerase chain reaction

Total RNA was extracted from testes using the FastPure Cell/Tissue Total RNA Isolation Kit V2 (RC112-01, Vazyme, Nanjing, China). This RNA was then reverse-transcribed into complementary DNA (cDNA) using the PrimeScript™ RT reagent Kit with gDNA Eraser (RR047A, Takara Bio, Inc., Kusatsu, Japan) to eliminate genomic DNA contamination. Subsequent reverse transcription quantitative polymerase chain reaction (RT-qPCR) analyses were performed using the Power SYBR® Green PCR Master Mix (4367659, Thermo Fisher Scientific) on an Applied Biosystems 7500 Real-Time PCR System. mRNA expression levels were normalized against the housekeeping gene *Actb* to ensure accuracy. Each experiment was replicated at least three times to ensure the reliability and reproducibility of the results. Detailed information on the primers used is provided in the Supplementary Information, specifically in Table S2.

### Co-immunoprecipitation and mass spectrometry

Testes from three 16 dpp mice were dissected and lysed in IP lysis buffer (P0013, Beyotime Biotechnology, Inc., China), supplemented with proteinase inhibitors to prevent degradation. The homogenates were centrifuged at 13,500 g for 15 minutes, and the supernatant was collected, with 40 µL being reserved as the input control. The remaining supernatant was incubated with either an NEDD8 antibody or a non-specific IgG antibody for 3 hours at 4 ℃ with continuous gentle rotation. Following incubation, pre-washed magnetic Protein A beads (10002D, Invitrogen, Waltham, MA, USA) were added and allowed to interact with the proteins overnight at 4 ℃ with continuous gentle rotation. The beads were then rinsed with PBS. Finally, the samples were boiled with 5 × SDS loading buffer to elute the proteins. For mass spectrometry analysis of NEDD8-IP proteins, the precipitated proteins were resolved on 4-20% NuPAGE gels (ThermoFisher Scientific), running 1h from the well, and then stained with SimplyBlue (LC6065, ThermoFisher Scientific) to facilitate in-gel digestion. The proteins in the gel were hydrolyzed with trypsin. In the liquid chromatography-tandem mass spectrometry (LC-MS/MS) analysis, the digested peptides were separated using a 120-minute gradient elution at a flow rate of 0.300 µL/min with the Thermo Vanquish Neo system, which was directly coupled to the Thermo Orbitrap Fusion Lumos mass spectrometer. An Acclaim PepMap RSLC column (75 µm ID, 250 mm length) was used as the analytical column. Mobile phase A consisted of 0.1% formic acid, while mobile phase B contained 80% acetonitrile and 0.1% formic acid. The Thermo Orbitrap Fusion Lumos mass spectrometer was set to data-dependent acquisition mode using Xcalibur 4.1.50 software, with a single full-scan mass spectrum in the Orbitrap (375-1500 m/z, 60,000 resolution) followed by subsequent data-dependent MS/MS scans. The MS/MS spectra from each LC-MS/MS run were searched against the selected database using the software Proteome Discovery (version 2.4). The normalized protein abundances were converted into a logarithmic scale, and differential analysis was performed using the R package limma (v3.50.3) 24. Proteins with an adjusted *P* value less than 0.05 and a fold change (IP/IgG) greater than 1 were identified as NEDD8-interacting candidates.

### Ubiquitinomics in wild type and *Nae1*-SKO testes

Proteins were mixed with five volumes of pre-chilled acetone and then precipitated at -20 ℃ for 2 hours to concentrate the protein content. Following precipitation, the protein samples were re-suspended in 200 mM Triethylammonium bicarbonate and subjected to ultrasonic dispersion to ensure thorough mixing. Trypsin was added at a ratio of 1:50 for overnight digestion at a temperature that facilitates enzyme activity. Prior to digestion, the samples were treated with 5 mM dithiothreitol for 30 minutes at 56 ℃ to reduce disulfide bonds, followed by the addition of 11 mM iodoacetamide for 15 minutes at room temperature in the dark to alkylate the cysteine residues, preventing reformation of disulfides. The resulting peptides were desalted using Strata X SPE columns (Phenomenex, Torrance, CA) and enriched by incubation with pre-washed antibody beads (PTM1104, PTM Bio LLC, Chicago, IL) in NETN buffer (100 mM NaCl, 1 mM EDTA, 50 mM Tris-HCl, 0.5% NP-40, pH 8.0) at 4 ℃ overnight with gentle shaking. The beads were then washed with NETN buffer to remove unbound peptides. Bound peptides were eluted from the beads using 0.1% trifluoroacetic acid, and the eluted fractions were combined and subjected to vacuum-drying to remove the solvent. For LC-MS/MS analysis, the peptides were further purified using C18 ZipTips (Millipore, Burlington, MA) according to the manufacturer's protocol. After preprocessing the raw ubiquitinomics data by eliminating missing values and omitting unnamed gene products, we applied a log2 transformation to the abundance values to ensure a normal distribution. To evaluate sample quality, we utilized boxplots to display the abundance distributions across various experimental groups. We further conducted repeatability analysis to gauge the reliability of our quantitative results by comparing the consistency between biological and technical replicates. Pearson correlation coefficients for the intensity values across all protein ubiquitination sites within the samples. These coefficients were then translated into a heatmap, offering a visual representation of the correlation structure within our dataset. Differential ubiquitination analysis was conducted with the limma package in R 24.

A linear model was applied to the expression data based on the experimental design matrix, which was then followed by empirical Bayes moderation of the standard errors. Ubiquitination sites that were significantly altered were pinpointed with criteria of *P*-value < 0.05 and absolute log2 fold change > 1 when comparing wild type (WT) and *Nae1*-SKO. The outcomes were graphed using volcano plots to illustrate the interplay between statistical significance and the extent of change. To gauge the broader effects on protein ubiquitination, we conducted analyses at both the site level and the gene level. In the gene-level analysis, the cumulative log2 fold changes were determined by aggregating the changes from individual sites within each gene, taking into account the total count of detected and significantly altered sites per protein. The functional annotation of the differentially ubiquitinated proteins was carried out with the clusterProfiler package with the org.Mm.eg.db annotation database 25.

### Quantification and statistical analysis

Statistical analyses were carried out using GraphPad Prism 9.0 (GraphPad Software, La Jolla, CA). At least three independent biological replicates were executed for all experiments, including histology, immunofluorescence, qPCR, Co-IP and western blot. Data were presented as mean and standard deviations. The statistical significance of difference was determined using an unpaired, two-tailed Student's t-test with a *P* < 0.05 being considered statistically significant. Specific *P* values and statistical tests were detailed in the main text.

## Results

### NAE1 is highly expressed since pachytene during spermatogenesis

**Figure 1:**
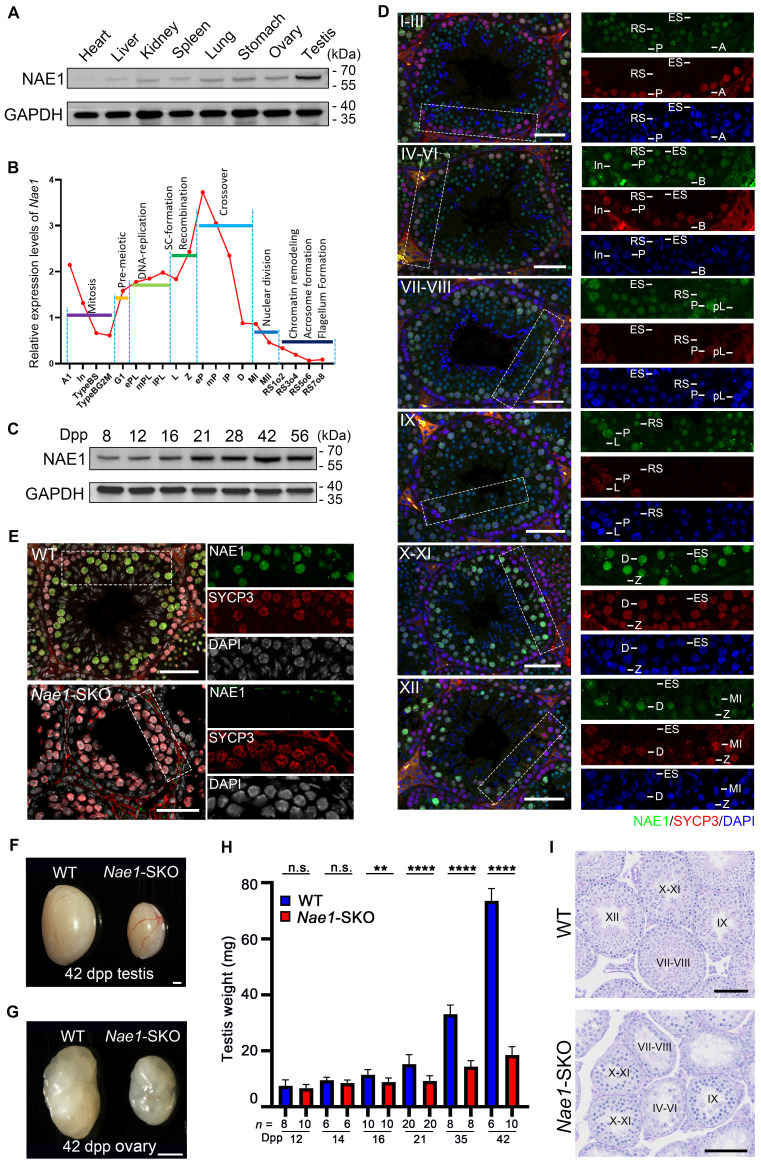
NAE1 is essential for mouse spermatogenesis and fertility. (A) Western blot analysis of NAE1 protein levels in tissue lysates from 6-week-old WT mice. (B) Relative mRNA expression levels of *Nae1* during mouse spermatogenesis at different stages of development. (C) Western blotting analysis of NAE1 protein levels in mouse testes at different ages. (D) Immunofluorescence co-staining of NAE1 and SYCP3 in 42 dpp WT mouse testes at different subdivision of the cycle of the mouse seminiferous epithelium. (E) Immunofluorescence co-staining of NAE1 and SYCP3 in 42 dpp WT and *Nae1*-SKO mouse testes. (F-G) Representative image showing the morphology of testes at 42 dpp (F) and ovaries at 42 dpp (G) derived from WT and *Nae1*-SKO mice. (H) Weight of testes derived from WT and *Nae1*-SKO mice at the indicated ages. Error bars indicate SEM. **P < 0.01, **** *P* < 0.0001 by two-tailed Student's t-test. n.s. means not significant. (I) Morphological analysis of the 42 dpp testes from WT and *Nae1*-SKO mice using PAS staining. Abbreviations: A1: type A1 spermatogonia; A: type A spermatogonia; In: intermediate spermatogonia; B: type B spermatogonia; BS: S stage type B spermatogonia; BG2: G2/M stage type B spermatogonia; G1: gap phase; pL: preleptotene; ePL: early preleptotene; mPL: mid preleptotene; lPL: late preleptotene; L: Leptotene; Z: Zygotene; P: pachytene; eP: early pachytene; mP: mid pachytene, lP: late pachytene; D: diplotene; MI: Metaphase I; MII: Metaphase II; RS: round spermatid. ES: elongated spermatid. Scale bar in (D) and (E) = 20 μm. Scale bar in (F) and (G) = 500 μm. Scale bar in (I) = 50 μm.

Mouse *Nae1*, or *Appbp1* (gene ID: 234664), is a homologue of the human *NAE1* (gene ID: 8883) and consists of 20 exons encoding a protein containing 534 amino acids. The sequence alignment indicated that NAE1 was a conserved protein expressed in various vertebrate species (Fig. S1A), and it possessed a highly conserved domain that interacting with the catalytic subunit UBA3 19 (Fig. S1B). NAE1 was ubiquitously expressed in organs, particularly in the testes (Fig. 1A). Transcriptome data from mouse germ cells 26 showed that *Nae1* expression levels began increasing during the leptotene/zygotene stage and remained relatively high throughout meiotic prophase I, especially during the pachytene stage (Fig. 1B). In mice, the first round of spermatogenesis is synchronous. To determine the temporal expression of NAE1, we analyzed testicular tissues collected on different post-natal days to capture the first wave of spermatogenesis (Fig. 1C). NAE1 protein was detected at 8 days postpartum (dpp) and continually increased in mouse testes since 12 dpp to sexual maturity stage, which is the developmental stage corresponding to the emergence of spermatocytes. Next, we co-stained paraffin-embedded sections of WT mice testes at different time points for NAE1 and SYCP3, a component of the meiotic chromosome lateral axis, marks spermatocytes in meiosis prophase I 27. The results showed that NAE1 signals was expressed in seminiferous tubule at 14 dpp. The number of NAE1-positive cells and fluorescence intensity gradually increased from 14 to 21 dpp (Fig. S1C). These results prompted further examination of the NAE1 expression patterns of spermatogenesis in the seminiferous tubules at different stages. Using SYCP3 and DAPI, the seminiferous tubule was divided into six phases: I-III, IV-VI, VII-VIII, IX, X-XI, and XII. Immunofluorescence staining showed that NAE1 was not expressed in spermatogonia (types A, In, and B), pre-leptotene, leptotene or zygotene spermatocytes. A weak NAE1 signal was observed in the nuclei of early-pachytene spermatocytes at phases I-III, and the signal intensity gradually increased in mid-pachytene during phases IV-VI. The signal reached its maximum value in the nuclei of late-pachytene at VII-IX and diplotene spermatocytes during phases X-XI, followed by gradual weakening from the MI stage in phases XII and complete disappearance in elongated spermatids (Fig. 1D). High expressions during pachytene/diplotene stage suggested that NAE1 may play an important role in the later stage of meiotic prophase I.

### Male and female mice with *Nae1*-deletion are infertile

Because early embryonic lethality is characteristic of *Nae1* homozygous null mice 28, we generated a mouse line with *Nae1* specifically deleted from the germ cells to explore the physiological role of NAE1 in spermatogenesis. Using CRISPR/Cas9-mediated editing, *loxP* sites were placed on both sides of the region from exons 4 to 11 to generate *Nae1*-flox mice. *Stra8*-Cre mice, in which recombination begins in type A1 spermatogonia (before meiosis) 29, were crossed with *Nae1*-flox mice to obtain *Nae1^fl/-^; Stra8-Cre* (*Nae1*-SKO) mice (Fig. S2A and B). NAE1 expression levels in *Nae1*-SKO testes were observed to be significantly lower than those in WT testes at 14 dpp (Fig. S2C). Then, we detected protein expression levels in the spermatocytes retrieved from FACS, which further verified the complete absence of NAE1 in *Nae1*-SKO spermatocytes (Fig. S2D). Additionally, immunofluorescence results confirmed the presence of NAE1 in SYCP3-positive cells in 6-week WT testes, whereas NAE1 signals were not detected in all SYCP3-positive spermatocytes in *Nae1*-SKO (Fig. 1E). This indicated that germ cell-specific *Nae1*-knockout mice were successfully established.

After mating with WT mice, both male and female *Nae1*-SKO mice were completely infertile. The testis size of the adult *Nae1*-SKO mice was significantly smaller than that of the littermate WT mice (Fig. 1F), and the adult *Nae1*-SKO female mouse ovary size was also smaller (Fig. 1G). Subsequently, we compared the testis weights of mice at different dpp and found that from 16 dpp onwards, the average testis weight of *Nae1*-SKO mice was significantly lower than that of the WT group (Fig. 1H). Furthermore, a single testis remained below 20mg at 42 dpp in *Nae1*-SKO mice. In contrast to the WT seminiferous tubules, which contained spermatogenic cells of various developmental stages, the *Nae1*-SKO testes exhibited a presence of spermatogonia and spermatocytes, without round or elongated spermatids observed (Fig. 1I ,S2E and 2F). The cells positive for PNA lectin, an acrosomal marker identifying spermatids and sperm 30, were absent from the seminiferous tubules of *Nae1*-SKO mice at 42 dpp (Fig. S2G). Additionally, the epididymal lumens of *Nae1*-SKO male mice were devoid of mature sperm. (Fig. S2E). These finding suggested sperm development in *Nae1*-SKO mice proceeded abnormally.

In addition, NAE1 ablation has been concomitantly associated with female infertility, which prompted us to perform histological analysis of ovaries. Initial observations indicated normal ovarian follicular reserves at 4 weeks post-natal in *Nae1*-SKO female mice. However, a subsequent evaluation at 18 weeks revealed a complete absence of ovarian follicles in in *Nae1*-SKO mice, implicating a potential dysregulation of NAE1-mediated pathways in the aberrant development of oocytes and premature ovarian insufficiency, culminating in female infertility (Fig. S3). Taken together, these results indicate that NAE1 is essential for male and female fertility.

### Single-cell transcriptomic data reveals that NAE1 depletion arrests meiosis and increases apoptosis

**Figure 2:**
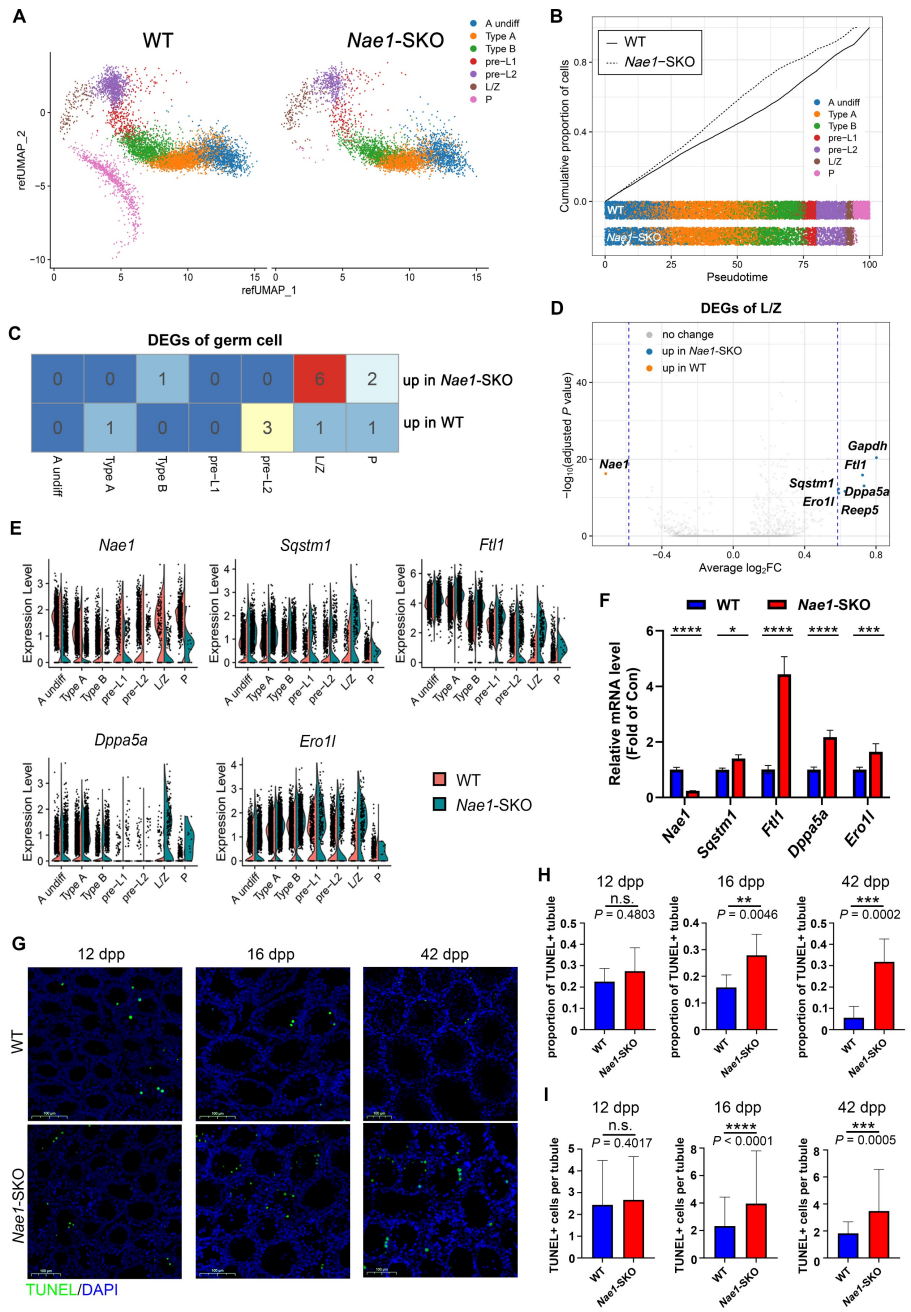
10 × scRNA-seq data showed an apoptosis increase in *Nae1*-SKO spermatocytes during meiosis. (A) Uniform manifold approximation and projection (UMAP) of profiles (dots) from sorted cells of murine testes of the indicated genotypes. Colored dots, cells collected from the indicated genotype with different colors showing different stages. (B) Cell ratio of germ cells of the indicated genotypes. Colors represent different cell populations. (C) Differentially expressed gene in germ cells of different stages compared between WT and *Nae1*-SKO mouse testes. (D) Volcano plots showing the verification of the differentially expressed genes between WT and *Nae1*-SKO leptotene/zygotene spermatocytes. (E) Expression levels and (F) qPCR validation of *Nae1* and apoptosis-related upregulated genes during leptotene/zygotene in *Nae1*-SKO mouse testes. (G) Immunofluorescence staining of TUNEL in WT and *Nae1*-SKO mouse testes. (H) and (I) Quantification of apoptotic tubules (TUNEL-positive cells). Error bars indicate SEM. *P < 0.05, **P < 0.01, ***P < 0.001, ****P < 0.0001 by two-tailed Student's t-test. n.s. means not significant. Abbreviations: A undiff: type A undifferentiated spermatogonia; Type A: type A spermatogonia; Type B: type B spermatogonia; pre-L: pre-leptotene; L/Z: leptotene/zygotene; P: pachytene; DEG: differentiated expressed genes. Scale bar in (G) = 100 μm.

To elucidate the specific stages at which NAE1-deficiency leads to abnormal spermatogenesis and to assess its impact on the transcriptional profile of germ cells, we performed 10 × scRNA-seq to elucidate the molecular characteristics and variations in germ cell subpopulations throughout spermatogenesis. Considering a significant difference in average testis weight was observed at 16 dpp (Fig. 1H), two mice at 16 dpp from each group, WT and *Nae1*-SKO, were selected for comparison. A total of 39,076 cells were isolated and uniform manifold approximation and projection (UMAP) analysis was conducted on the subset datasets 31 (Table S3). The uniform manifold approximation and projection (UMAP) analysis outlined a continuous trajectory of germ cell development characterized by selected markers identifying major testicular cell types, like type A undifferentiated spermatogonia (*Gfra1*^+^), type A differentiated spermatogonia (*Stra8*^+^), type B differentiated spermatogonia (*Esx1*^+^), leptotene spermatocytes (*Dmc1*^+^), zygotene spermatocytes (*Meiob*^+^), and pachytene spermatocytes (*Piwil1*^+^) (Fig. 2A, S4A and B).

Upon comparing the cell numbers between the two groups, a general decline in spermatocytes was observed in *Nae1*-SKO group (Fig. S4C). Of note, there is exceedingly small number of cells capable of reaching the pachytene stage in *Nae1*-SKO mice compared with WT suggesting that the spermatogenesis was arrested at this stage (Fig. 2B and S4C). Subsequently, we engaged in an analysis of the DEGs within germ cells at different stages. The overall transcriptional differences were relatively minor between WT and *Nae1*-SKO (Fig. 2C). The transcription level of *Nae1* was significantly decreased in *Nae1*-SKO testes, confirming the reliability of 10 × scRNA-seq results (Fig. 2C, D, and E). However, what caught our attention was a marked escalation in the expression of six genes, which was discerned within the *Nae1*-SKO mouse during the leptotene/zygotene (L/Z) phase (Fig. 2C and D). Among them, the expression of *Sqstm1*^32^, *Ftl1*^33^, *Dppa5a*^34^ and *Ero1l* 35, which have been corroborated for their nexus to apoptotic mechanisms, were observed to exhibit a pronounced elevation in *Nae1*-SKO mice, especially during prophase I of meiosis (Fig. 2E). The subsequent qPCR assays confirmed the increase in their transcriptional levels (Fig. 2F). Thereafter, TUNEL assay demonstrated increased apoptosis in *Nae1*-SKO testes since 16 dpp compared with WT testes (Fig. 2G), characterized by an increased proportion of TUNEL-positive tubule, along with a significant increase in the number of TUNEL-positive cells per tubule (Fig. 2H and I). Most apoptotic cells were found to be predominantly spermatocytes. This observation suggested that the absence of NAE1 induced the upregulation of a plethora of apoptosis-related genes in leptotene/zygotene spermatocyte, culminating in apoptosis during meiosis.

### *Nae1*-SKO spermatocyte development is arrested at late pachytene stage with abnormal synapsis at XY chromosomes

**Figure 3:**
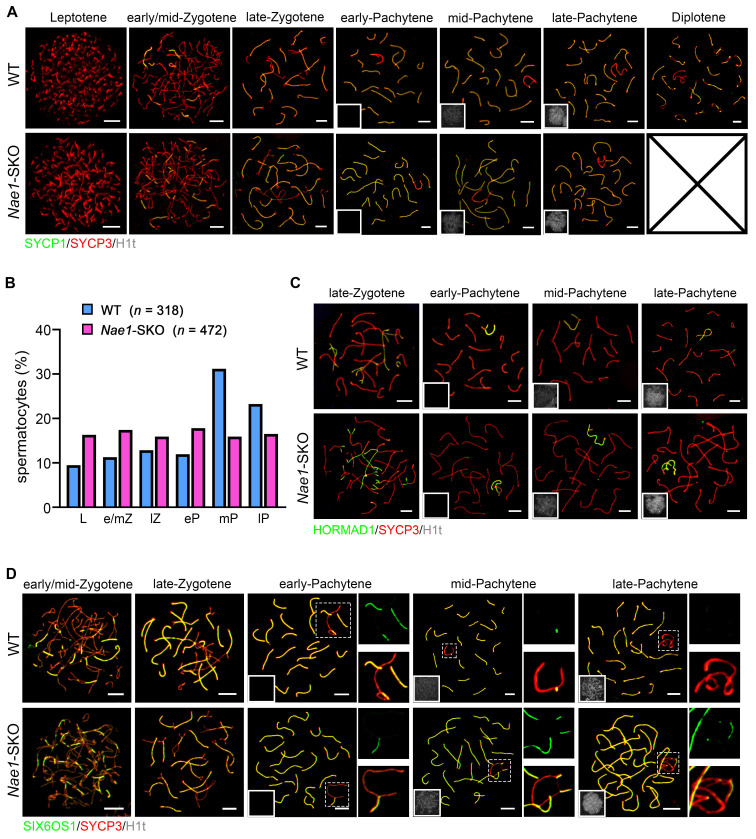
NAE1 deletion leads to meiosis arrest at pachytene stage. (A) Immunofluorescence co-staining of SYCP1 and SYCP3 on surface-spread spermatocytes in WT and *Nae1*-SKO mouse testes from leptotene to diplotene stage. (B) Percentage of spermatocytes in leptotene to pachytene stage from 56 dpp WT and *Nae1*-SKO mouse testes. (C) Immunofluorescence co-staining of HORMAD1 and SYCP3 on surface-spread spermatocytes in WT and *Nae1*-SKO mouse testes from late-zygotene to late-pachytene stage. (D) Immunofluorescence co-staining of SIX6OS1 and SYCP3 on surface-spread spermatocytes in WT and *Nae1*-SKO mouse testes from early/mid-zygotene to late-pachytene stage. Right panels of pachytene spermatocytes show enlarged insets of XY body area. Miniaturised H1t signal of the corresponding cell is shown in the bottom left corner of immunofluorescence images of pachytene spermatocytes. Abbreviations: L: Leptotene; e/mZ: early/mid zygotene; lZ: late zygotene; eP: early pachytene; mP: mid pachytene, lP: late pachytene. Scale bar in immunofluorescence images = 5 μm.

To further confirm that the meiotic substage was primarily affected by *Nae1* deletion, we assessed H1t, a marker observed since mid-pachytene stage 27 (Fig. S5A). The decreased in numbers of H1t-positive spermatocytes further confirmed a meiosis I prophase developmental failure. To further determine the arrest of prophase I substage in *Nae1*-SKO spermatocytes, we co-stained SYCP3 with SYCP1 (a marker of homologue synapsis) and H1t on nuclear spreads of spermatocytes using 35 dpp testes from* Nae1*-SKO and littermate WT mice 36 (Fig. 3A). According to SYCP3/SYCP1 signal and chromosomal morphology change, meiotic prophase I stages could be divided into leptotene, zygotene, pachytene, and diplotene. Specifically, pachytene was separated to early-pachytene, mid-pachytene, and late-pachytene based on H1t signal intensity. All stages of meiotic prophase I were observed in the nuclei of WT spermatocytes, with most at pachytene (51.1%). SYCP1 signals appeared at zygotene stage and were located at the midline of SYCP3-stained axes in every autosome at pachytene, and finally, autosomes began desynapsis at the diplotene stage. A similar cell cycle progression pattern was observed in *Nae1*-SKO mice; however, only spermatocytes from leptotene to pachytene stages were observed. We further quantitatively compared the progression of meiotic prophase I in pubertal 35 dpp testes from WT nuclei versus those in* Nae1*-SKO nuclei. A significantly higher prevalence of leptotene (9.5% vs. 16.3%), zygotene (24.2% vs. 33.3%) and early pachytene cells (11.9% vs. 17.8%) was observed in *Nae1*-SKO, while there was a notably lower prevalence of mid-pachytene (31.2% vs. 15.9%) and late-pachytene (23.2% vs. 16.5%) cells. Furthermore, *Nae1*-SKO mice contained no diplotene cells, indicating a developmental delay with a late-pachytene arrest during spermatogenesis (Fig. 3B).

To investigate the defective meiotic progression in *Nae1*-SKO mice, we initially explored whether NAE1 plays a role in synapsis. The synapsis is actually the process of synaptonemal complex (SC) assembly; hence, we examined SYCP1, SC transverse filament, and HORMAD1, unsynapsed chromosomal axes 37 using spermatocyte spreading (Fig. 3A and C). We found efficient autosome pairing and synapsis in both WT and *Nae1*-SKO mice, which was confirmed in the result of co-staining SIX6OS1, the SC central element 38 (Fig. 3D). Conversely, the XY chromosomes exhibited behaviors different from autosomes in pachytene *Nae1*-SKO spermatocytes. Synaptic maturation on XY chromosomes was disordered, as manifested by a prolonged fluorescence signal (Fig. 3D). These results indicate that *Nae1* deletion leads to normal autosomes synapsis but defects in XY maturation.

### Meiotic recombination defects with class I crossovers are impaired by *Nae1* deficiency

**Figure 4:**
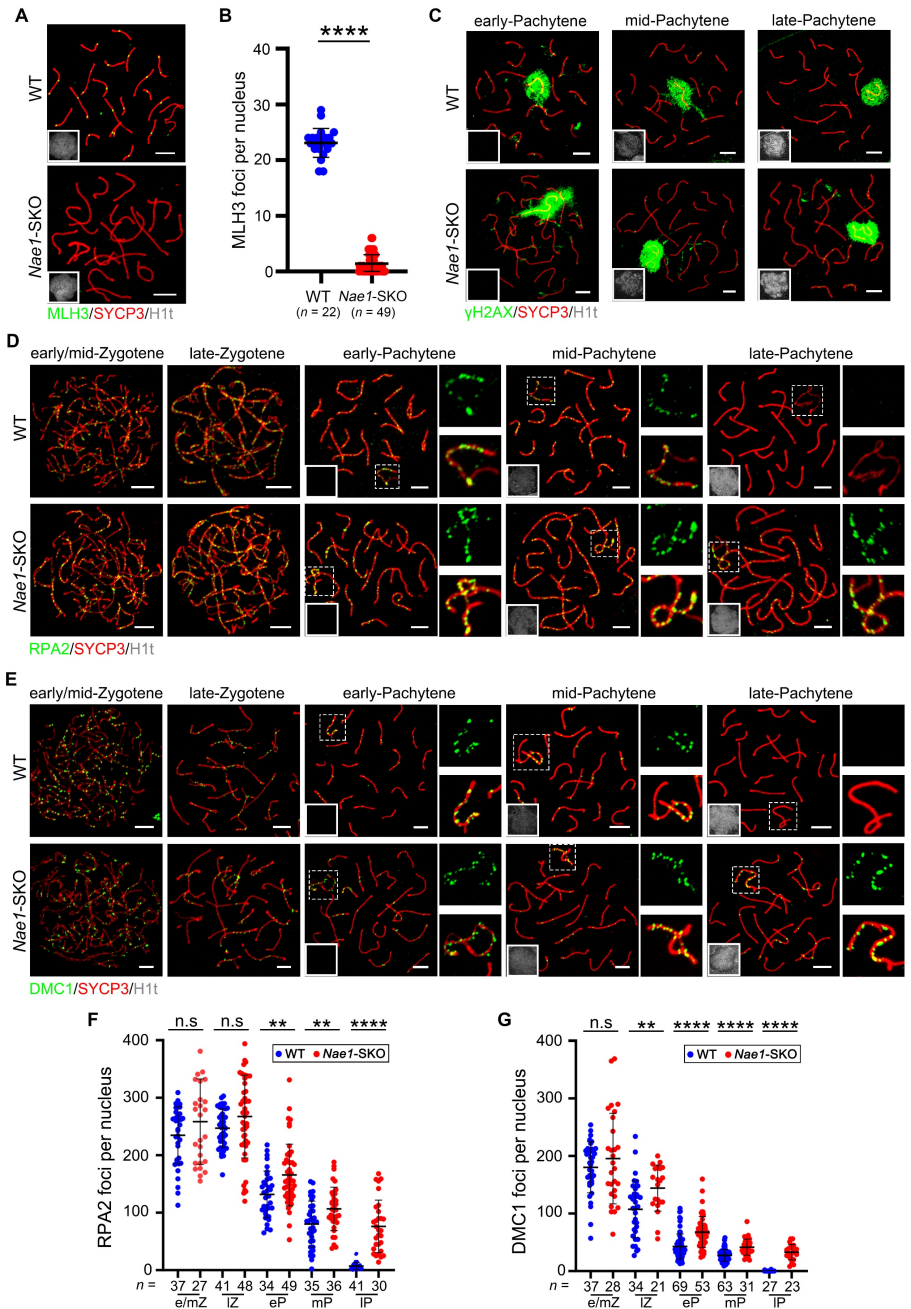
Meiotic recombination defect with class I crossovers impaired in *Nae1*-SKO mice. (A) Immunofluorescence co-staining of MLH3 and SYCP3 on surface-spread spermatocytes in WT and *Nae1*-SKO mouse testes in late pachytene stage. (B) The quantification of the number of MLH3 foci associated with the chromosome axes per nucleus. (C) Immunofluorescence co-staining of γH2AX and SYCP3 on surface-spread spermatocytes in WT and *Nae1*-SKO mouse testes during pachytene stage. Immunofluorescence co-staining of RPA2 (D) and DMC1 (E) with SYCP3 on surface-spread spermatocytes in WT and *Nae1*-SKO mouse testes from early/mid-zygotene to late-pachytene stage. Right panels of pachytene spermatocytes show enlarged insets of XY body area. The quantification of the number of RPA2 (F) and DMC1 (G) foci associated with the autosome axes per nucleus. Miniaturised H1t signal of the corresponding cell is shown in the bottom left corner of immunofluorescence images of pachytene spermatocytes. Abbreviations: e/mZ: early/mid zygotene; lZ: late zygotene; eP: early pachytene; mP: mid pachytene, lP: late pachytene. Scale bar in immunofluorescence images = 5 μm. (B), (F) and (G) *n* shows the number of spermatocytes analyzed for each stage. Error bars indicate SEM. **P < 0.01, ****P < 0.0001 by two-tailed Student's t-test. n.s. means not significant.

Considering the impaired meiotic progression, it is plausible to infer potential defects in meiotic recombination. So, we investigated MLH3, a component of late recombination nodules MutLγ that serves as a class I crossover marker 12. The results revealed the absence of MLH3 foci in *Nae1*-SKO late-pachytene spermatocytes (1.4 ± 1.6 in *n* = 49 in *Nae1*-SKO nuclei vs. 23.1 ± 2.6 in *n* = 22 in WT nuclei; *P* < 0.0001), suggesting that crossover formation was also impaired (Fig. 4A and B).

In WT males, CDK2 foci are typically observed at telomeres, at one or two interstitial sites on each synapsed bivalent region, and on the synapsed portions of the X and Y chromosomes 39. However, the non-telomeric CDK2 had essentially disappeared at the pachytene stage in* Nae1*-SKO spermatocytes, with the foci number reduced to 0.3 ± 1.4 per cell in* Nae1*-SKO spermatocytes (*n* = 79), which was markedly fewer than the 21.3 ± 3.4 per cell (*n* = 77) in the WT spermatocytes. (Fig. S5B and C). In summary, meiotic recombination and formation of class I crossovers were compromised by NAE1-deficiency.

Meiotic crossover recombination is contingent upon the successful completion of programmed DSB, which are often indicated by phosphorylated H2AX (γH2AX) 40. In WT spermatocytes, a low level of γH2AX were observed in chromatin flares on synapsed autosomes during early pachytene, signifying some unrepaired DSBs. As most DSBs were repaired, there was a subsequent loss of autosomal γH2AX flares in mid-pachytene and beyond. In *Nae1*-SKO spermatocytes, the γH2AX signal was detected at leptotene and zygotene stages, similar to that observed in WT spermatocytes (Fig. S5D), suggesting a normal initiation of DSB. Notably, γH2AX was observed on *Nae1*-SKO autosomes in the mid-pachytene and beyond stages, instead of limited convergence on the XY bodies, as shown in WT samples (Fig. 4C). This prompted that NAE1 depletion might lead to DSB repair failure. To confirm this, we performed immunofluorescence on RPA2, which was also examined to evaluate DSB repair 41. In zygotene stage, WT and* Nae1*-SKO spermatocytes showed a comparable number of foci. However, the RPA2 protein on the chromosomal axis of *Nae1*-SKO spermatocytes presented a delayed turnover, with foci persisting in *Nae1*-SKO nuclei since early pachytene (165.6 ± 53.7, *n* = 49 in *Nae1*-SKO vs. 131.8 ± 40.6, *n* = 34 in WT, respectively; *P* = 0.0026), mid-pachytene (106.7 ± 37.8, *n* = 36 in *Nae1*-SKO vs. 80.3 ± 39.8, *n* = 35 in WT, respectively;* P* = 0.0056), and late pachytene (76.3 ± 45.6, *n* = 30 in *Nae1*-SKO vs 7.0 ± 6.5, *n* = 41 in WT, respectively; *P* < 0.0001) (Fig. 4D and F). This observation was consistent with the findings for γH2AX, suggesting deficient DSB repair after NAE1 deletion. RPA complex coat 3' single strand DNA that is resected during DSB, and subsequently, RAD51 and DMC1 recombinases facilitate homology search and strand invasion after replacing RPA2 42, 43. To further elucidate the recombination defects in *Nae1*-SKO spermatocytes, we investigated the localization of the meiosis-specific recombinase DMC1 and RAD51 (Fig. 4E and S6A). The numbers of DMC1 foci at early/mid-zygotene were comparable between WT and *Nae1*-SKO mice, suggesting the replacement of RPA complexes occurred normally in *Nae1*-SKO spermatocytes. However, a high number of DMC1 foci persisted throughout late-zygotene to late-pachytene (Fig. 4G). Consistent with the changes in DMC1, the dynamics of RAD51 foci in *Nae1*-SKO nuclei were also similar. (Fig. S6B). These kinetics of DMC1 and RAD51 indicate a delay in turnover of recombinases, suggesting a crossover differentiation defect due to abnormal stabilization of strand-invasion intermediates. Beyond that, high levels of DSB repair markers and recombinase on XY chromosomes were maintained throughout the pachytene stage in *Nae1*-SKO mice (Fig. S6C, D and E). These results indicate aberrant DSB repair in XY chromosomes of* Nae1*-SKO spermatocytes, which is consistent with abnormal XY maturation detected by SC markers.

### Depolymerization of recombination intermediates and crossover differentiation are abnormal in *Nae1*-SKO mice

**Figure 5:**
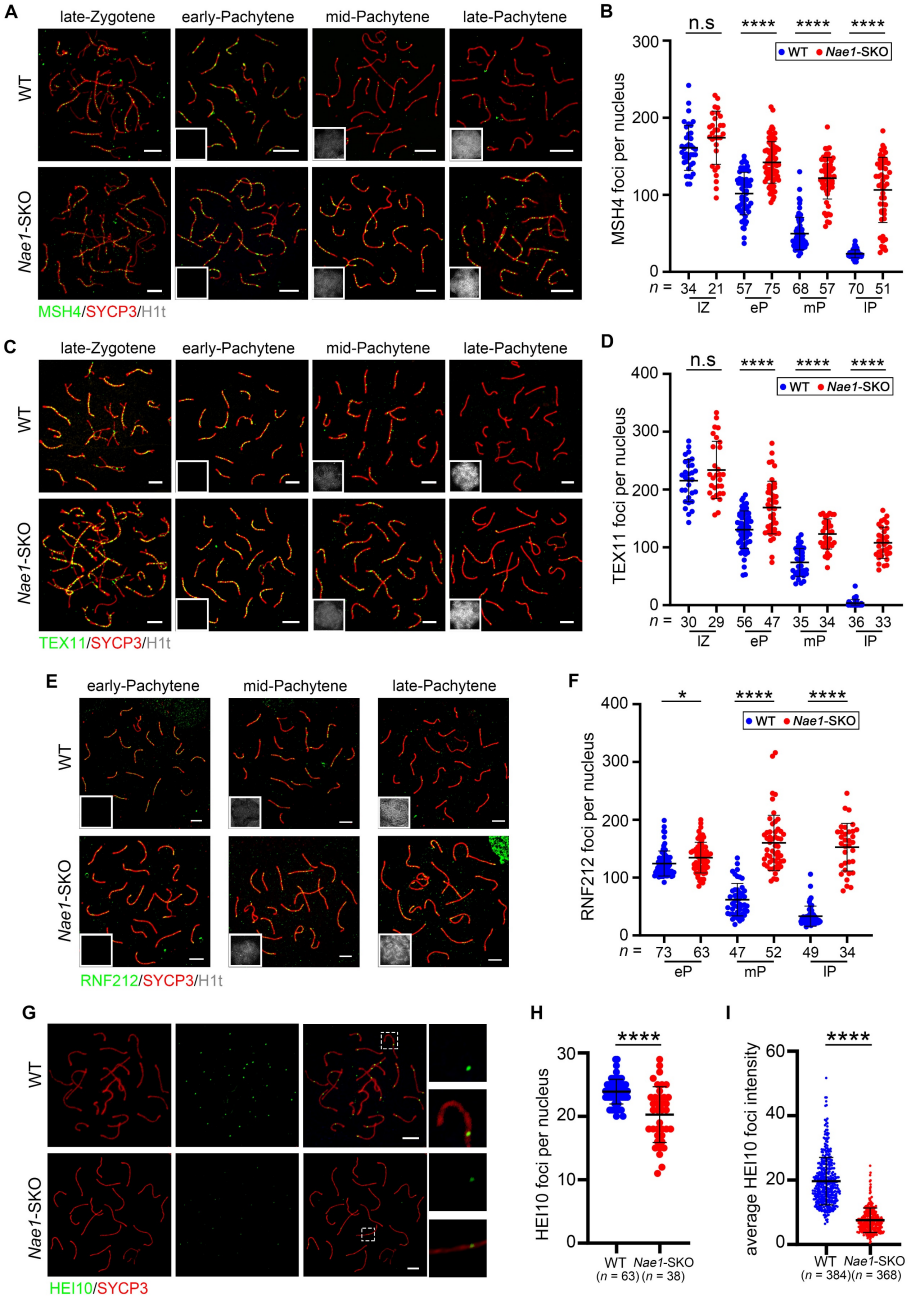
NAE1 affects differential stabilization of crossover sites to regulate meiotic recombination. Immunofluorescence co-staining of MSH4 (A) and TEX11 (C) with SYCP3 on surface-spread spermatocytes in WT and *Nae1*-SKO mice testes from late-zygotene to late-pachytene stage. The quantification of the number of MSH4 (B) and TEX11 (D) foci associated with the chromosome axes per nucleus. Immunofluorescence co-staining of RNF212 (E) and HEI10 (G) with SYCP3 on surface-spread spermatocytes in WT and *Nae1*-SKO mice testes during pachytene stage. The quantification of the number of RNF212 (F) and HEI10 (H) foci associated with the chromosome axes per nucleus. (I) The quantification of HEI10 signal intensities. Miniaturised H1t signal of the corresponding cell is shown in the bottom left corner of immunofluorescence images of pachytene spermatocytes. Abbreviations: lZ: late zygotene; eP: early pachytene; mP: mid pachytene, lP: late pachytene. Scale bar in immunofluorescence images = 5 μm. (B), (D), (F), (H) and (I) *n* shows the number of spermatocytes analyzed for each stage. Error bars indicate SEM. *P < 0.05, ****P < 0.0001 by two-tailed Student's t-test. n.s. means not significant.

The MZIP2-TEX11-SPO16 complex plays a pivotal role in binding and stabilizing early recombination intermediates, including strand-invasion intermediates and D-loops. Following this, the MSH4-MSH5 complex acts to promote and stabilize the branched recombination intermediates, such as dHJ 44. To understand whether the recombination intermediate was stable in the absence of NAE1, we examined related markers. In WT mice, MSH4 foci were localized as numerous foci along the synaptonemal complexes, with a sharp decreasing trend from early to late pachytene. However, the MSH4 signals on the chromosomal axes in *Nae1*-SKO spermatocyte nuclei, compared with WT samples, remained high throughout early pachytene (142.0 ± 27.0, *n* = 75 in *Nae1*-SKO vs. 101.6 ± 27.6, *n* = 57 in WT, respectively), mid-pachytene (121.6 ± 27.0, *n* = 57 in *Nae1*-SKO vs. 49.7 ± 21.1, *n* = 68 in WT, respectively), and late pachytene (106.3 ± 42.4, *n* = 51 in *Nae1*-SKO vs. 23.5 ± 5.1, *n* = 70 in WT, respectively), showing significant differences (*P* < 0.0001) (Fig. 5A and B). Moreover, we examined another component of ZMM proteins, TEX11 45. Similarly, the chromosomal dynamics of TEX11 were severely altered (Fig. 5C). The difference in the number of TEX11 foci between *Nae1*-SKO and WT mice was first observed in early pachytene (168.9 ± 45.5, *n* = 47 vs. 130.6 ± 32.5, *n* = 56, respectively), and persisted at the synapsed regions of the homologues during mid-pachytene (123.1 ± 26.2,* n* = 34 in *Nae1*-SKO vs. 73.9 ± 24.1,* n* = 35 in WT, respectively), and late pachytene (107.9 ± 27.1, *n* = 33 in *Nae1*-SKO vs. 3.5 ± 6.5, *n* = 36 in WT, respectively). (Fig. 5D). Together, the persistence of MSH4 and TEX11 foci during pachytene suggests abnormal intermediate stabilization, causing failed crossover differentiation.

The stabilization of MutSγ and TEX11 at recombination sites is regulated by RNF212 and HEI10, and crucially determining the crossover fate. The equilibrium of their activities is fundamental to the temporal changes of MutSγ in spermatocytes 14, 46. The prolonged persistence of recombination markers is closely associated with, and likely contingent upon the sustained retention of RNF212 on chromosomes. We confirmed numerous RNF212 foci in *Nae1*-SKO spermatocytes during the pachytene stage; specifically, 134.6 ± 26.5, 160.3 ± 47.7, and 152.7 ± 41.2 foci were observed in early-, mid-, and late-pachytene, respectively, in *Nae1*-SKO nuclei, all showing significant differences when compared with those in WT mice (Fig. 5E and F). In contrast, HEI10 was thought to limit the colocalisation of RNF212 with MutSγ-associated recombination sites. The number of HEI10 foci was significantly decreased in *Nae1*-SKO compared with WT (20.3 ± 4.4, *n* = 38 in *Nae1*-SKO vs. 23.9 ± 1.9, *n* = 63 in WT, respectively; *P* < 0.0001) during pachytene (Fig. 5G and H). Meanwhile, *Nae1*-SKO pachytene spermatocytes contained weaker HEI10 foci, with less than half of the signal intensity of the WT pachytene spermatocytes (Fig. 5I). Altogether, these observations suggest that the absence of *Nae1* affect the stabilization of crossover sites along the autosomal axis.

### NAE1-mediated neddylation promotes the cellular protein ubiquitination during meiosis

**Figure 6:**
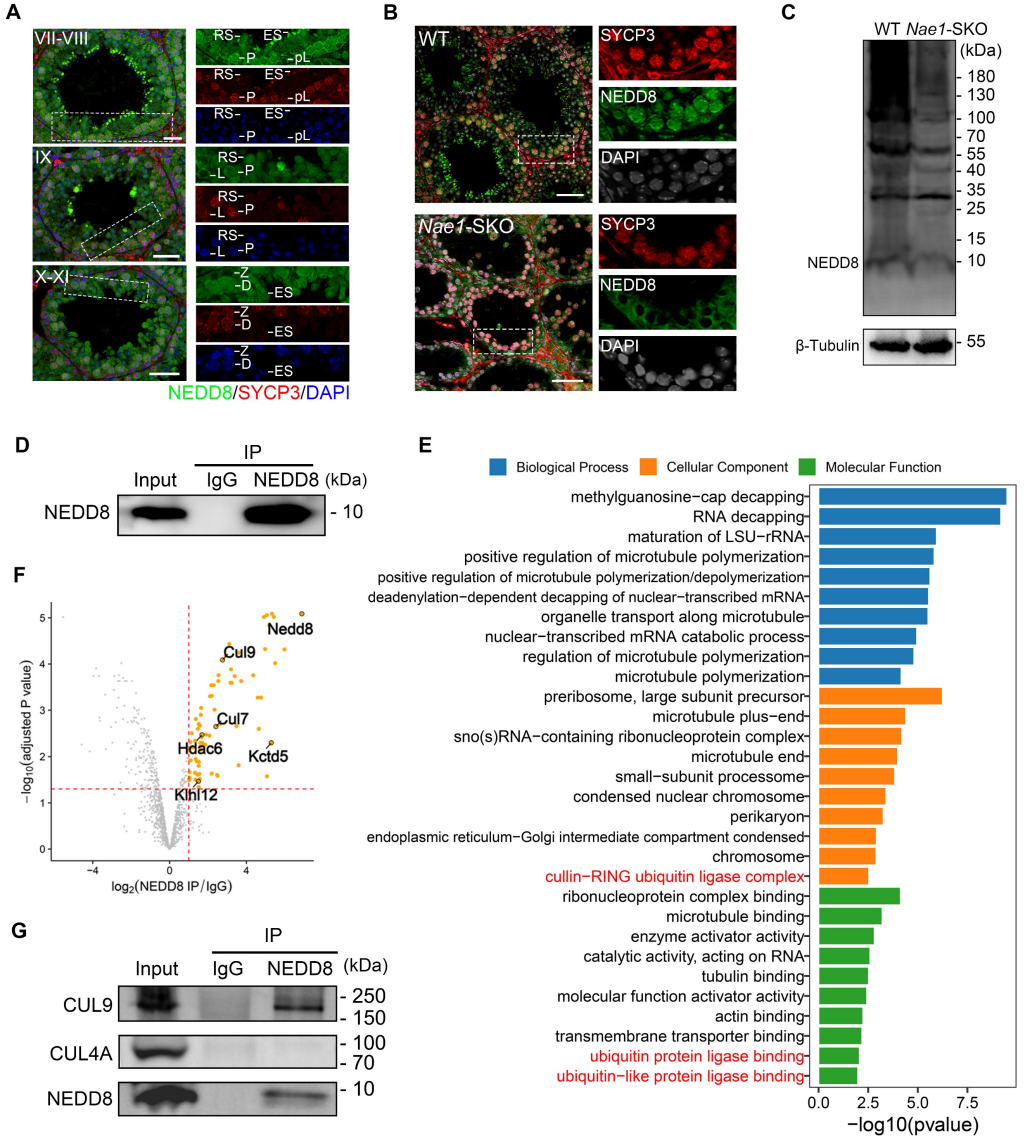
Ubiquitin E3 ligase CUL9 is involved in NAE1-activated neddylation regulating meiosis. (A) Immunofluorescence co-staining of NEDD8 and SYCP3 in 42 dpp WT mouse testes at different subdivision of the cycle of the mouse seminiferous epithelium. (B) Immunofluorescence co-staining of NEDD8 and SYCP3 in WT and *Nae1*-SKO mouse testes. (C) Western blot analysis of NEDD8 protein levels in WT and *Nae1*-SKO testes. (D) Western blot detection of immunoprecipitation by antibodies of IgG and NEDD8. (E) Bar plot showing the upregulated GO terms enriched compared with IgG. The length of the bar represents the gene count. The color represents the different functions. (F) Volcano plots showing NEDD8-interacting partners identified by IP-MS. (G) Immunoblots of immunoprecipitation experiments from testis extracts of adult mice. Abbreviations: pL: preleptotene; L: Leptotene; Z: Zygotene; P: pachytene; D: diplotene; RS: round spermatid. ES: elongated spermatid. Scale bar in (A) = 20 μm. Scale bar in (B) = 50 μm.

As previously mentioned, NAE1 is the regulatory subunit of the neddylation-activating enzyme E1. NEDD8 expression patterns at various stages of spermatogenesis were scrutinized to determine ascertain its susceptibility to NAE1 modulation. Consistent with NAE1, NEDD8 was also expressed in the nuclei of early-pachytene spermatocytes in phases I-III, and the signal gradually increased until diplotene spermatocytes reached phases X-XI (Fig. 6A and S6F). This indicated that the expression trend of NAE1 and neddylation modification during mouse meiotic prophase I exhibited a congruence. Subsequently, to clarify whether *Nae1* deletion influenced neddylation, NEDD8 expression was assessed and compared in WT and* Nae1*-SKO mice. Immunofluorescence results confirmed the presence of NEDD8 in the nuclei of SYCP3-positive cells in 6-week WT testes, whereas NEDD8 signals were not detected in any the nuclei of SYCP3-positive spermatocytes in *Nae1*-SKO testes (Fig. 6B). Consistently, western blotting results showed that the expression level of NEDD8 in *Nae1*-SKO mice testis was also significantly lower than that in WT mice at 14 dpp (Fig. 6C). This underscored the perturbation in NAE1-mediated neddylation pathways.

To identify the proteins that contribute to spermatogenesis regulation of NAE1-mediated neddylation, we thus conducted an IP-MS analysis utilizing a NEDD8 antibody (Fig. 6D). This approach was taken to broadly identify NEDD8 interactomes in 16 dpp testes. In total, 79 putative candidates NEDD8 interacting proteins were identified (Table S4). GO analysis of the candidate NEDD8 interactors highlighted an enrichment of terms associated with Cullin-RING ubiquitin ligase complex, ubiquitin protein ligase binding, and ubiquitin-like protein ligase binding (Fig. 6E). Remarkably, the Cullin family proteins are considered the primary physiological targets of NEDD8. Among the our NEDD8 IP-MS results, the Cullin family proteins CUL7 and CUL9 were detected with a significant presence of unique peptides (Fig. 6F), which serves to further validate the potential role of NAE1-activated neddylation in testes. Furthermore, Co-Immunoprecipitation (Co-IP) by NEDD8 antibody indicated that CUL9 interacted with NEDD8 in mouse testis (Fig. 6G). In contrast, CUL4A, which was previously confirmed to be associated with meiosis 47, 48, was detected do not associate with NEDD8 in testis (Fig. 6G). This indicated that NAE1-mediated neddylation may specifically regulate CUL9 function in meiosis, thereby influencing the process of spermatogenesis.

**Figure 7:**
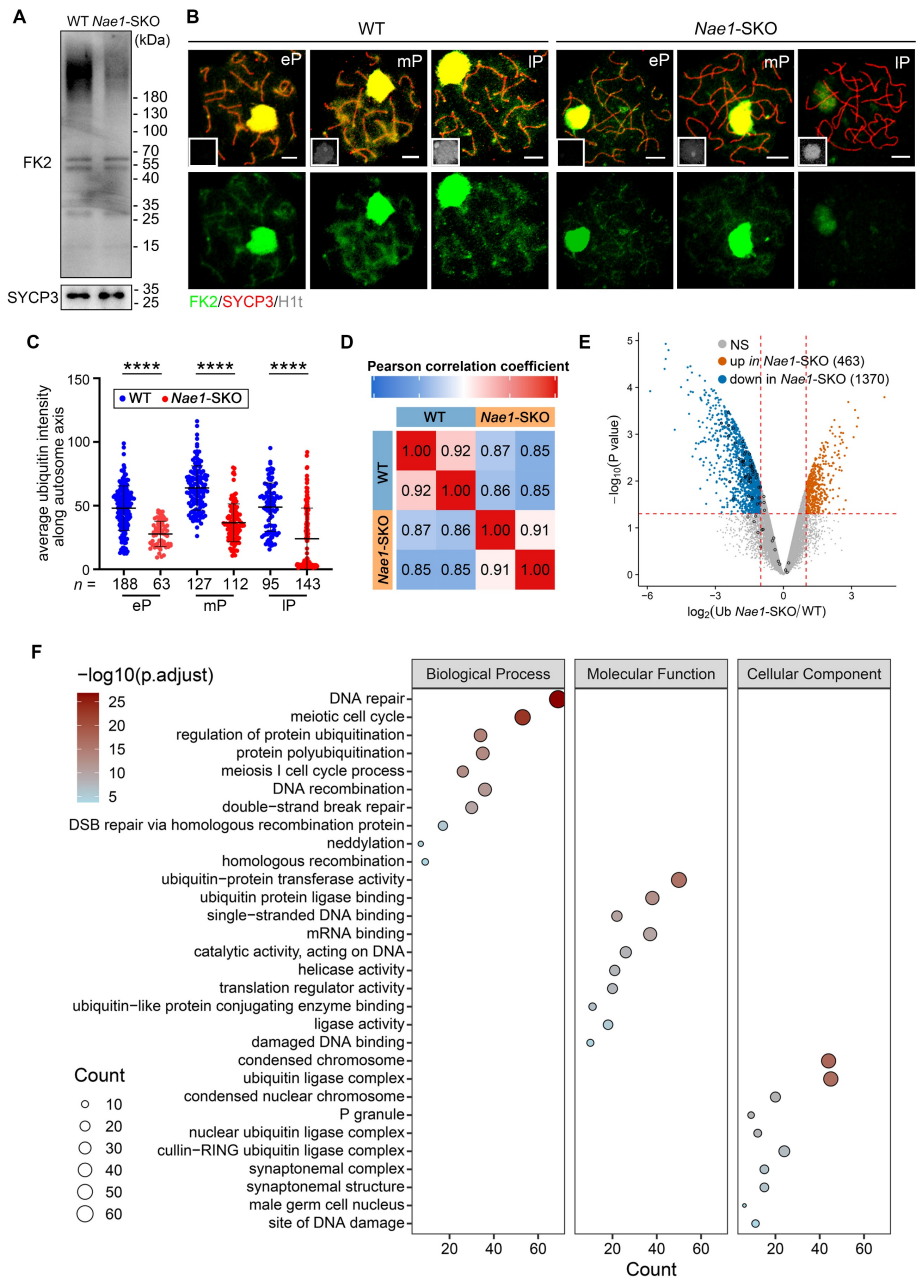
NAE1-activated neddylation regulates meiosis by modulating ubiquitination. (A) Western blot analysis of FK2 protein levels in WT and *Nae1*-SKO testes. (B) Immunofluorescence co-staining of FK2 and SYCP3 on surface-spread spermatocytes in WT and *Nae1*-SKO mice testes from early-pachytene to late-pachytene stage. (C) The quantification of average ubiquitin signal intensities per autosome axis. (D) Heatmap showing the Pearson correlation coefficient of ubiquitinomics profiles between intragroup repetition in WT and *Nae1*-SKO testes. (E) Volcano plots showing the verification of the differentially expressed ubiquitination-binding sites between WT and *Nae1*-SKO testes identified by ubiquitinomics data. (F) Bubble chart showing the downregulated GO terms enriched in *Nae1*-SKO testes compared with WT. The bubble size represents the gene count. The color intensity represents the average expression level of the indicated genes. Miniaturised H1t signal of the corresponding cell is shown in the bottom left corner of immunofluorescence images of pachytene spermatocytes. Abbreviations: eP: early pachytene; mP: mid pachytene, lP: late pachytene. Scale bar in (B) = 5 μm. (C) *n* shows the number of spermatocytes analyzed for each stage. Error bars indicate SEM. ****P < 0.0001 by two-tailed Student's t-test.

CRLs exerts its effects by modulating the levels of intracellular ubiquitination. To clarify impact of NAE1-mediated neddylation on cellular protein ubiquitination during spermatogenesis, we used the anti-ubiquitin antibody FK2, which recognises both mono and polyubiquitinating conjugates 49. Western blotting results indicated that ubiquitination levels were reduced in the testes at 14 dpp after *Nae1* deletion (Fig. 7A). In pursuit of a more nuanced view of ubiquitination during meiosis, we performed spermatocyte nuclear surface spreading and immunofluorescence staining (Fig. 7B). A congruent decline in ubiquitination levels was noted within pachytene spermatocytes. Upon quantification of the fluorescence intensity, a pronounced reduction in ubiquitination across the *Nae1*-SKO pachytene spermatocytes was discernible (Fig. S6G). Subsequently, a comparative analysis of fluorescence intensity along each autosome axis was performed, which corroborated the overall trend (Fig. 7C). These observations suggest that the absence of *Nae1* leads to a substantial reduction in ubiquitination levels during pachytene spermatocytes.

To elucidate the underlying mechanisms of neddylation regulating ubiquitination in the germ cells, we conducted ubiquitinomics analysis on WT and *Nae1*-SKO testes to facilitated the identification of ubiquitination sites within the 16 dpp testes. Pearson's correlation analysis was employed to confirm the high consistency among the replicate samples (Fig. 7D). Upon thorough examination, a total of 13,410 ubiquitination sites across 4,832 genes exhibited significant alterations in ubiquitination levels subsequent to NAE1 deletion (Table S5). In this cohort, 463 sites were observed to have an upregulation and 1,370 sites demonstrated a downregulation (Fig. 7E). Employing GO analysis, we further scrutinized changes in *Nae1*-SKO testes compared to WT. The analysis unveiled that the enriched biological process of down-regulated ubiquitination-binding genes in *Nae1*-SKO testes were associated with DNA repair, meiotic cell cycle process, DNA recombination, and DSB repair, indicating a disruption of normal meiosis in *Nae1*-SKO mice. The enriched molecular function and cellular component of down-regulated ubiquitination-binding genes in *Nae1*-SKO testes further suggests the crosstalk between neddylation and ubiquitination (Fig. 7F). Taken together, above analyses collectively revealed that NAE1-mediated neddylation promotes spermatocytes ubiquitination, thereby regulating homologous recombination.

## Discussion

Spermatogenesis, characterized by highly regulated meiotic division, is regulated by a suite of proteins that are procedurally activated. The significance of ubiquitination, a key PTM in eukaryotes is becoming increasingly recognized for its significance in critical aspects of meiotic development during spermatogenesis 50, 51. Neddylation, a PTM closely akin to ubiquitination, follows a distinct functional trajectory compared to the ubiquitin pathway, yielding different biological consequences 52. E1 activating enzymes set off a downstream cascade reaction that profoundly affects a multitude of cellular substrates. Consequently, the functional versatility and scope of these substrates place E1 enzymes as regulators in a broad spectrum of cellular processes 53. NAE1 has been associated with a range of pathologies and metabolic irregularities both prenatally and postnatally 28, 54, and has been established as a significant, independent prognostic factor for postoperative relapse 55.

**Figure 8:**
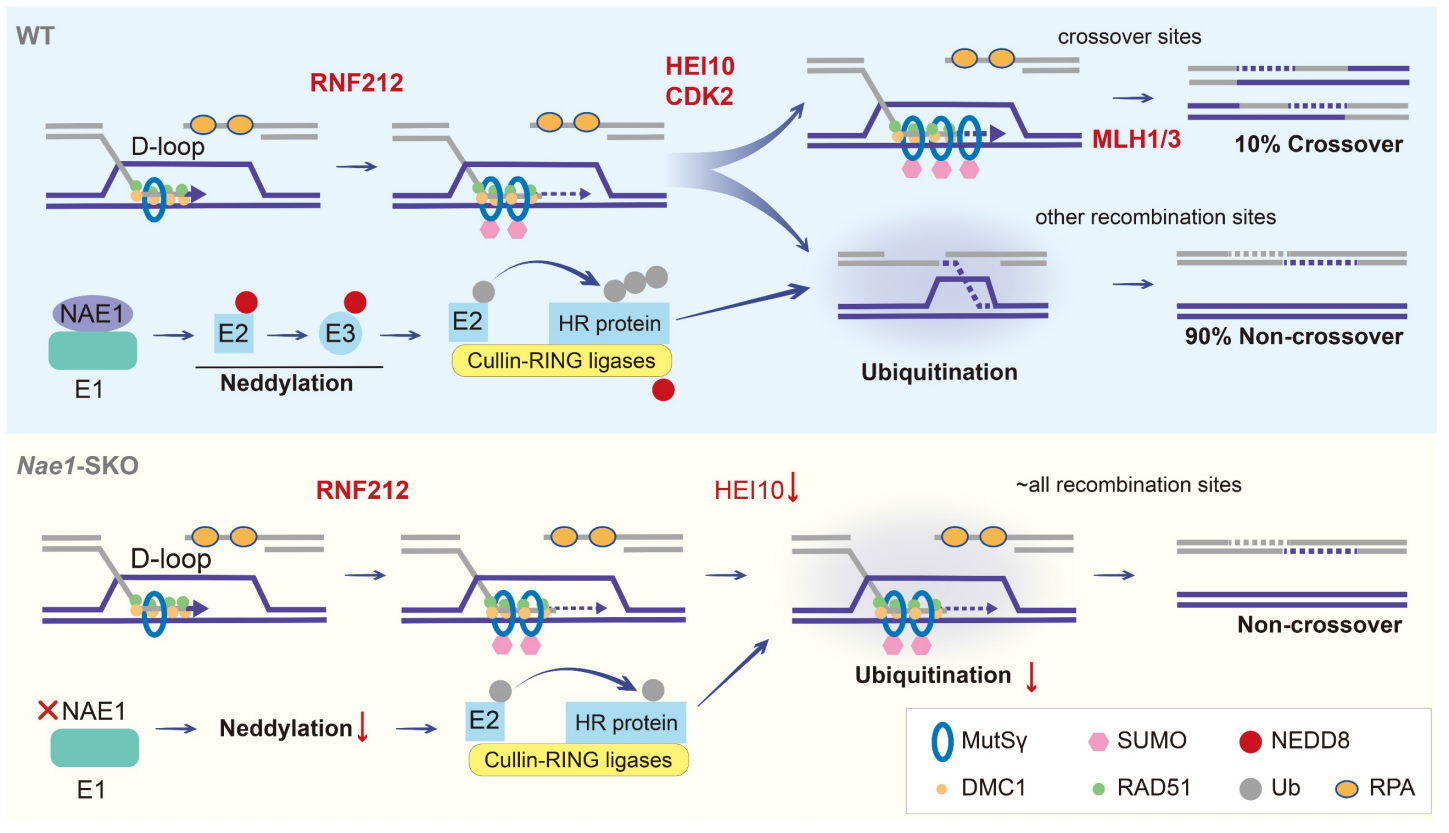
Models of crossover maturation and defects in *Nae1*-SKO mice. Under the effect of RAD51 and DMC1 recombinases, the 3' single-stranded DNA engages in DNA repair by utilizing the invaded homologous chromosome as a template, thereby culminating in the formation of unstable D-loop structures. Subsequently, the MutSγ complex, which functions in a manner akin to a sliding clamp, and the SUMOylation mediated by RNF212, collaborate in a positive feedback loop to stabilize the D-loop structures that constitute the recombination intermediates. The majority of these intermediates are subjected to neddylation, orchestrated by activating enzyme E1, which consisting of NAE1. NAE1-activated neddylation modulates the CRL complex to ubiquitinate a plethora of DNA homologous recombination-associated proteins. This regulation of homologue recombination protein stability facilitates the repair of recombination sites in a non-crossover configuration. Approximately 10% of these recombination intermediates, under the synergistic action of proteins such as HEI10 and CDK2, maintain the stability of RNF212/MutSγ, shielding them from interference and allowing for their accumulation, which results in the formation of stable dHJ structures. These structures are ultimately resolved into crossovers through the function of the MutLγ complex. In the absence of NAE1-mediated neddylation, there is a marked reduction in the ubiquitination capacity of the CRL complex. This leads to the continuous presence of RNF212 and MutSγ complexes on all recombination intermediates throughout the pachytene stage, culminating in a failure of crossover differentiation. DSBs are repaired in the form of non-crossover exclusively when RNF212 and MutSγ are shed.

This study demonstrated that the deletion of NAE1 led to neddylation defect, which seriously affected spermatogenesis. *Nae1*-SKO male mice showed a late-pachytene arrest in spermatocytes, resulting in infertility. Integrated analyses indicated that NAE1-activated neddylation may regulate the ubiquitination on the pachytene stage autosomal axis, which is similar to the sites where other PTMs regulate the process of meiosis 14, 46. Abnormal ubiquitination levels result in the persistence of MutSγ at chromosomes. This persistence leads to the formation of aberrant recombination intermediate structures, which affect crossover differentiation. Furthermore, a significant number of potential recombination sites are unable to recruit MutLγ/CDK2 to form crossover (Fig. 8). Our analysis identifies neddylation as a previously undiscovered PTM involved in meiosis and provides a new insight into the process of homologous recombination.

During spermatogenesis, selection and formation of crossovers during meiosis are of utmost importance, as they are crucial for ensuring the genetic diversity of offspring. Several molecular determinants have been implicated in the resolution of crossover fates, and the related knockout mouse models showed meiotic progression is feasible until metaphase I in male mice 46, 56-58, whereas NAE1-deficient male mice have arrested development at the late pachytene stage and manifest substantial apoptotic activity. The underlying causes may be attributed to the multifaceted role of NAE1-mediated neddylation, which is not only involved in crossover determination but also plays an integral part in a variety of other cellular pathways. In our dataset, NEDD8-associated proteins were found to be enriched in the Cullin family, specifically CUL9, within testicular tissue. When NEDD8 attaches to Cullin proteins, the inhibitory binding by Cullin-associated and neddylation-dissociated-1 (CAND1) is disrupted, and CRLs are activated 59. It is imperative to note that, CRL family represents the most extensive group of E3 ubiquitin ligases, tasked with orchestrating the ubiquitination of approximately 20% of cellular proteins destined for degradation through the ubiquitin-proteasome system 60. Our study elucidates that a neddylation-ubiquitination system (NUS) network being active as sequential modification during meiosis, which is based on the Cullin family.

In addition,* Nae1*-SKO female mice did not produce any pups after crossing with WT males. Paraffin section showed that primordial, primary, and secondary follicles could be observed in the ovaries of 4-week female mice. This indicated that meiosis reached diplotene stage with NAE1 deletion, which differed from late-pachytene arrest in male phenotype. This difference is understandable, as the pachytene check point in female mice is less stringent than in males, as evidenced by the observation of follicles development in adult *Mlh1*^-/-^, *Mlh3*^-/-^,* Prr19*^-/-^,* Rnf212*^-/-^ mice 56, 57, 61, 62. In addition, only cortical stroma without any follicles remains in the 18-week and beyond stage *Nae1*-SKO ovaries. This suggests that a decline in NAE1-mediated neddylation may lead to the overactivation of the primordial follicle and even apoptosis, which could lead to premature consumption of follicle pool. These results suggest that NAE1 plays multiple roles in oogenesis and is indispensable for female fertility.

## Conclusion

We propose that NAE1-mediated neddylation regulates ubiquitination during meiosis and is therefore involved in the stabilization of recombinant proteins related to crossover differentiation. Our investigation unveiled a previously unknown mechanism wherein the neddylation-ubiquitination system (NUS) modulates the orchestration of homologous recombination.

## Supplementary Material

Supplementary figures and tables.

## Abbreviations

PTM: post-translational modifications
DSB: double-strand break
NUS: neddylation-ubiquitination system
dHJ: double Holliday junction
NEDD8: Neural precursor cell-expressed developmentally down-regulated 8
CRL: Cullin-RING ligases
HE: hematoxylin and Eosin
PAS: Periodic Acid-Schiff
DAPI: 4,6-diamidino-2-phenylindole
FACS: fluorescence-activated cell sorting
DEG: differentially expressed gene
GO: Gene Ontology
RT-qPCR: reverse transcription quantitative polymerase chain reaction
IP‑MS: Immunoprecipitation‑mass spectrometry
LC-MS/MS: liquid chromatography-tandem mass spectrometry
dpp: days postpartum
WT: wild type
*Nae1*-SKO: *Nae1^fl/-^; Stra8-Cre*
UMAP: uniform manifold approximation and projection
