# Supplementary material for: NAE1-mediated neddylation coordinates ubiquitination regulation of meiotic recombination during spermatogenesis
Source: Theranostics. 2025 Feb 10;15(7):3122–42. doi: 10.7150/thno.107843 (PMC11898277; doi:10.7150/thno.107843)
Supplement: Supplementary file 1 — Supplementary figures and tables. [file thnov15p3122s1.zip › Supplementary_Data-revision.pdf]

SUPPLEMENTARY INFORMATION FOR

NAE1-mediated neddylation coordinates ubiquitination regulation of meiotic recombination during spermatogenesis

Yu Xi[1,3†], Chenjia Gong[1,2,4,5,6†], Zhe Zhang[1,3†], Feiyin Zhu[1,2,4,5,6,7†], Ying Zhang[1,2,4,5,6],

Yanlin Tang[1,3], Liying Yan[1,2,4,5,6], Hui Jiang[1,3,8,9,10*], Jie Qiao[1,2,4,5,6,7*], Qiang Liu[1,2,4,5,6*]

[†]Contributed equally

***Correspondence:**

Qiang Liu (http://orcid.org/0000-0002-8032-5985). E-mail: lqiang1210@bjmu.edu.cn

Jie Qiao (http://orcid.org/0000-0003-2126-1376). E-mail: jie.qiao@263.net

Hui Jiang (http://orcid.org/0000-0002-6256-2733). E-mail: jianghui@bjmu.edu.cn

This PDF file includes:

Supplementary Figures S1-S7

Supplementary Tables S1-S2

A

	HUMAN	MOUSE	RAT	CHIMPANZEE	BOVIN	CAT	RHESUS MACAUQE	GOAT	DOG	HORSE
HUMAN	1									
MOUSE	97.0037%	1								
RAT	96.8165%	97.9401%	1							
CHIMPANZEE	97.3783%	95.1311%	95.1311%	1						
BOVIN	97.7528%	97.191%	96.4419%	95.6929%	1					
CAT	95.1311%	94.0075%	94.0075%	92.0074%	95.6929%	1				
RHESUS MACAUQE	99.4382%	97.5655%	97.191%	97.191%	98.3146%	95.5056%	1			
GOAT	97.451%	97.0588%	96.2745%	95.8824%	99.0196%	94.5098%	98.0392%	1		
DOG	96.0076%	95.057%	95.057%	93.9163%	95.8175%	96.7681%	96.3878%	93.7255%	1	
HORSE	97.6608%	97.076%	96.4912%	95.5166%	97.8558%	94.7368%	98.2456%	93.5294%	94.152%	1

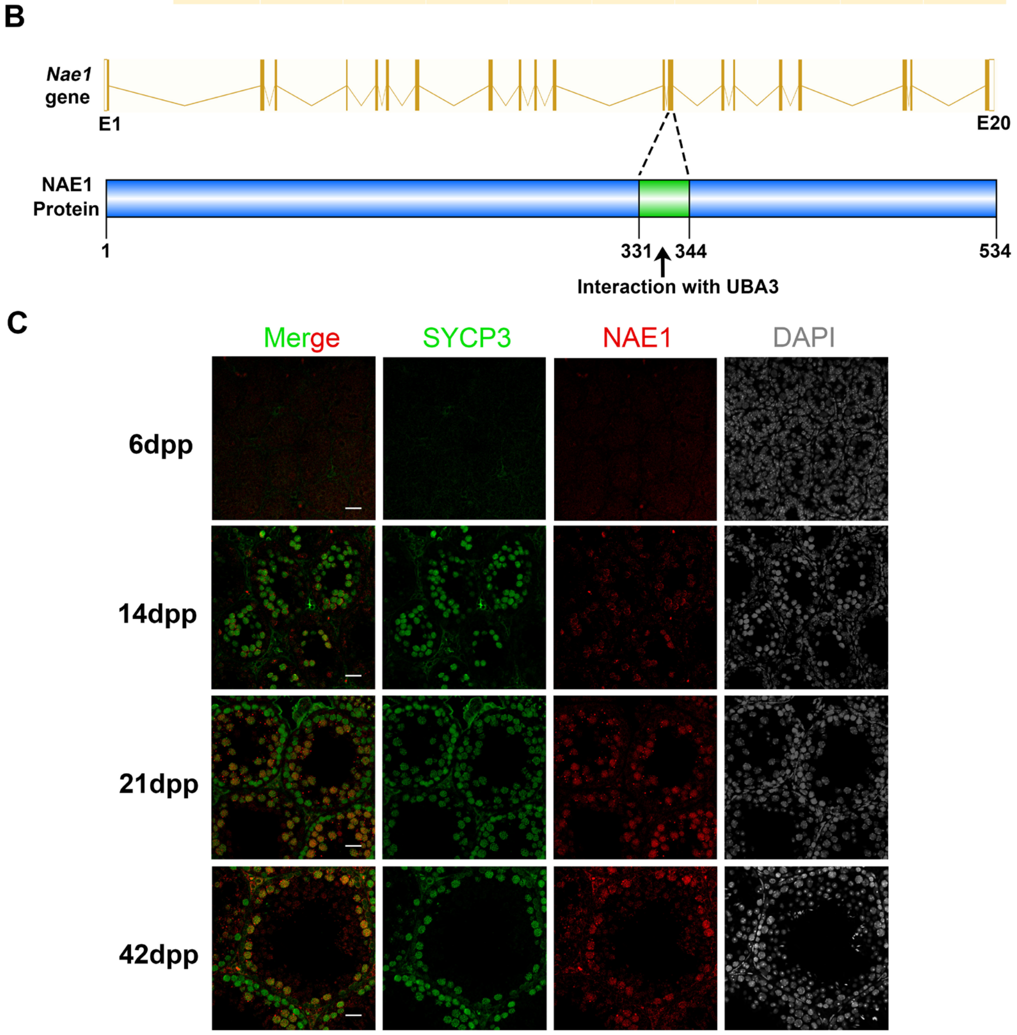

Figure S1. NAE1 is highly conserved in different species
(A) Sequence alignment of NAE1 amino acids among 10 species.
(B) Representation of NAE1 with its known domains.
(C) Immunofluorescence staining of NAE1 and SYCP3 in WT mouse testes at 6, 14, 21, and 42 days postpartum. Scale bar in (C) = 10 μm.

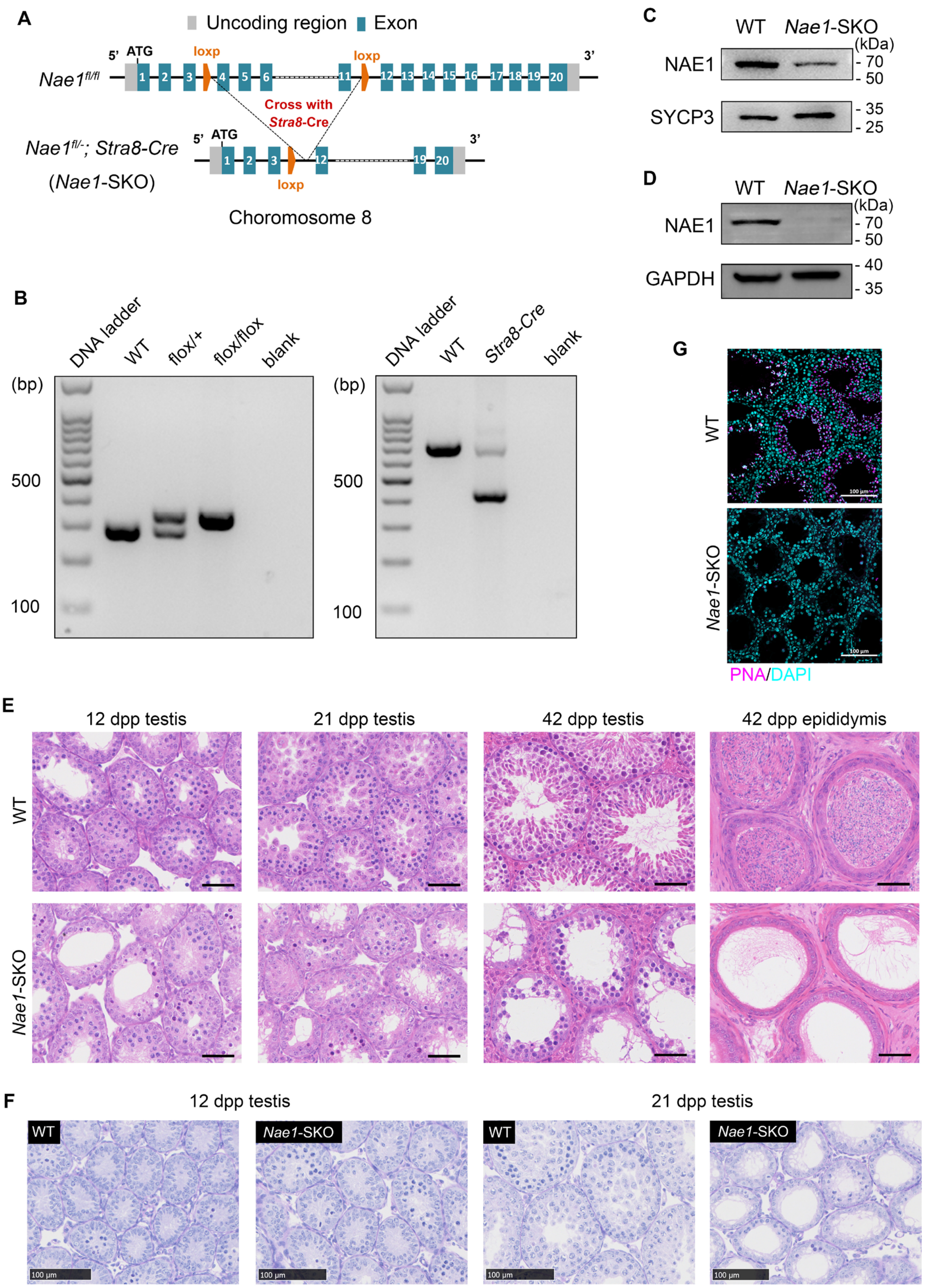

Figure S2. *Nae1* conditional knockout genotype identification and *Nae1*-SKO mouse characterization

(A) Schematic representation of the *Nae1* conditional targeting construct.

(B) Genotype identification of *Nae1* conditional knockout mice using the *Nae1*-flox and *Stra8-Cre* primers. The primer sequences are listed in Supplementary TableS2.

(C) Western blot analysis of NAE1 protein levels in WT and *Nae1*-SKO testes.

(D) Western blot analysis of NAE1 protein levels in WT and *Nae1*-SKO spermatocytes.

(E) Morphological analysis of the testes and epididymis from WT and *Nae1*-SKO mice using HE staining.

(F) Morphological analysis of the testes from WT and *Nae1*-SKO mice using PAS staining.

(G) Immunofluorescence staining of PNA in WT and *Nae1*-SKO mouse testes.

Scale bar in (E) = 50 μm, (F) and (G) = 100 μm.

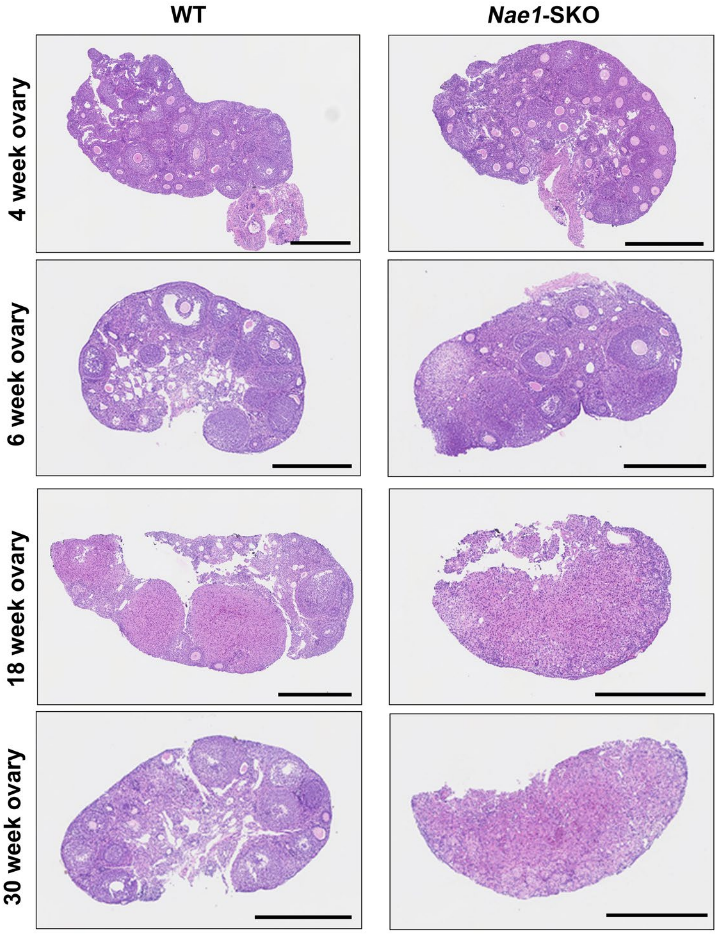

Figure S3. The morphology of ovaries derived from WT and *Nae1*-SKO mice.

Scale bar = 50 μm.

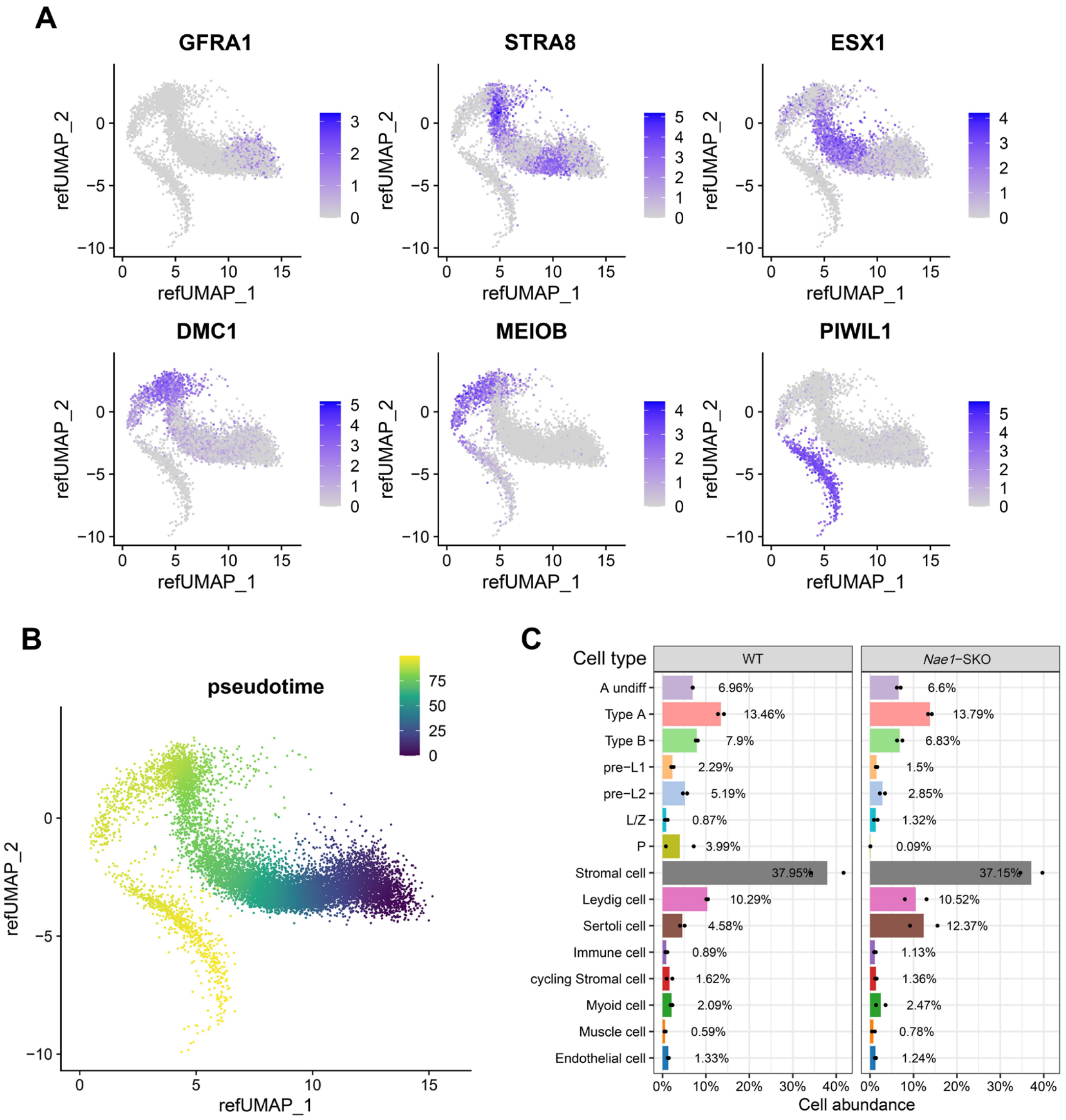

Figure S4. 10 × scRNA-seq data clustering and proportion characteristics of WT and *Nae1*-SKO testes

(A) Expression patterns of selected markers identifying major testicular germ cell types, projected on the UMAP plot.

(B) Developmental trajectory along spermatogenesis.

(C) Cell counts for each stage from murine testes of the indicated genotypes related to panel.

Abbreviations: A undiff: type A undifferentiated spermatogonia; Type A: type A spermatogonia; Type B: type B spermatogonia; pre-L: pre-leptotene; L/Z: leptotene/zygotene; P: pachytene.

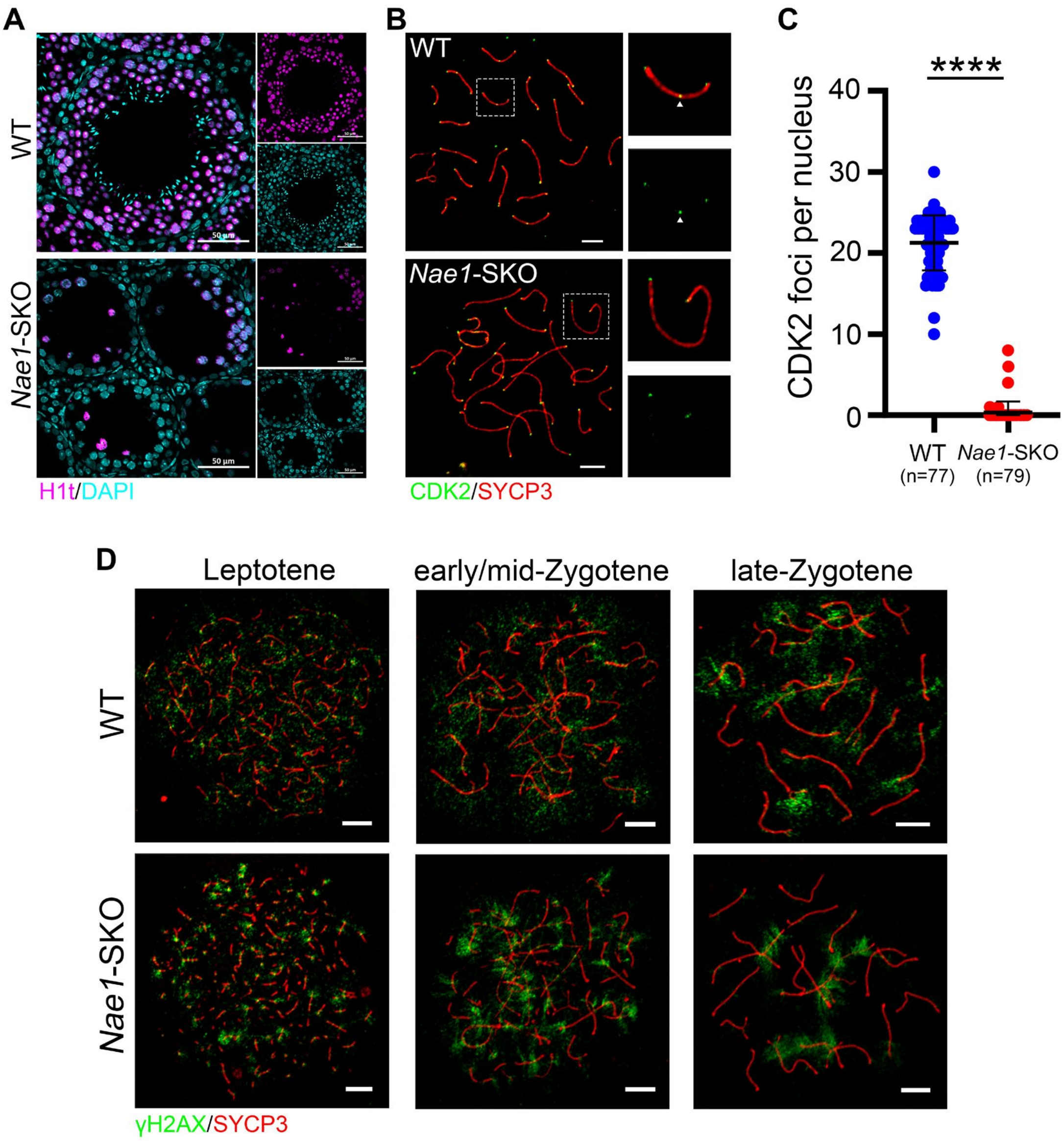

Figure S5. NAE1 deletion leads to pachytene arrest

(A) Immunofluorescence staining of H1t in WT and *Nae1*-SKO mouse testes.

(B) Immunofluorescence co-staining of CDK2 and SYCP3 on surface-spread spermatocytes in WT and *Nae1*-SKO mice testes in pachytene stage. Right panels show enlarged insets of non-telomeric CDK2 signal foci (pointed by the triangular arrow) located at chromosome axis.

(C) The quantification of the number of CDK2 foci associated with the chromosome axes per nucleus.

(D) Immunofluorescence co-staining of γH2AX and SYCP3 on surface-spread spermatocytes in WT and *Nae1*-SKO mice testes from leptotene to late-zygotene stage.

Scale bar in (A) = 50 μm. (B) and (D) = 5 μm.

(C) *n* shows the number of spermatocytes analyzed . Error bars indicate SEM. ****$P < 0.0001$ by two-tailed Student's t-test.

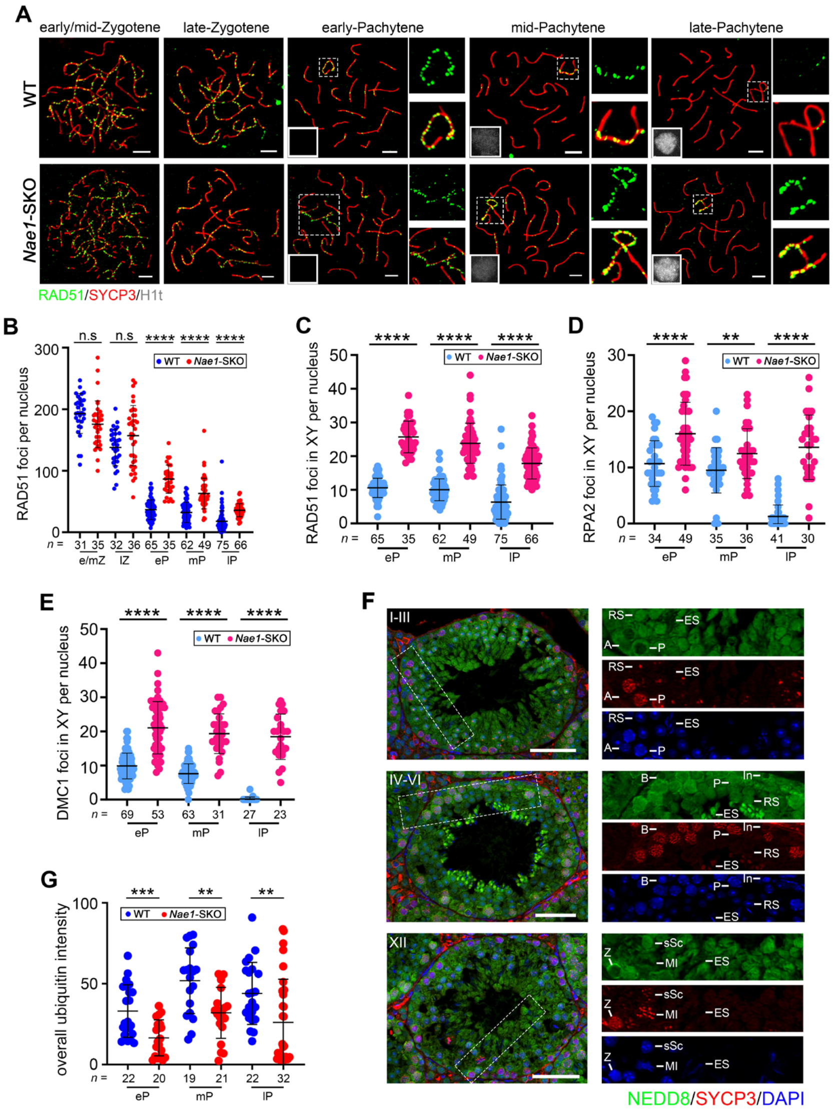

Figure S6. NAE1-deletion affects DSB repair and neddylation

(A) Immunofluorescence co-staining of RAD51 with SYCP3 on surface-spread spermatocytes in WT and *Nae1*-SKO mice testes from early/mid-zygotene to late-pachytene stage. Right panels of pachytene spermatocytes show enlarged insets of XY body area.

(B) The quantification of the number of RAD51 foci associated with the autosome axes per nucleus

(C) The quantification of the number of RAD51 foci on XY chromosomes axes per nucleus.

The quantification of the number of RPA2 (D) and DMC1 (E) foci on XY chromosomes axes per nucleus.

(F) Immunofluorescence co-staining of NEDD8 and SYCP3 in 42 dpp WT mouse testes at different subdivision of the cycle of the mouse seminiferous epithelium.

(G) The quantification of overall ubiquitin signal intensities outside sex body.

Miniaturised H1t signal of the corresponding cell is shown in the bottom left corner of immunofluorescence images of pachytene spermatocytes.

Abbreviations: A: type A spermatogonia; In: intermediate spermatogonia; B: type B spermatogonia; e/mZ: early/mid zygotene; lZ: late zygotene; eP: early pachytene; mP: mid pachytene, lP: late pachytene; sSc: secondary spermatocyte; pL: preleptotene; L: Leptotene; Z: Zygotene; P: pachytene; MI: Metaphase I; RS: round spermatid. ES: elongated spermatid.

Scale bar in (A) = 5 µm. (F) = 20 µm.

(B), (C), (D), (E) and (G) *n* shows the number of spermatocytes analyzed. Error bars indicate SEM. ***P* < 0.01, ****P* < 0.001, *****P* < 0.0001 by two-tailed Student's t-test. n.s. means not significant.

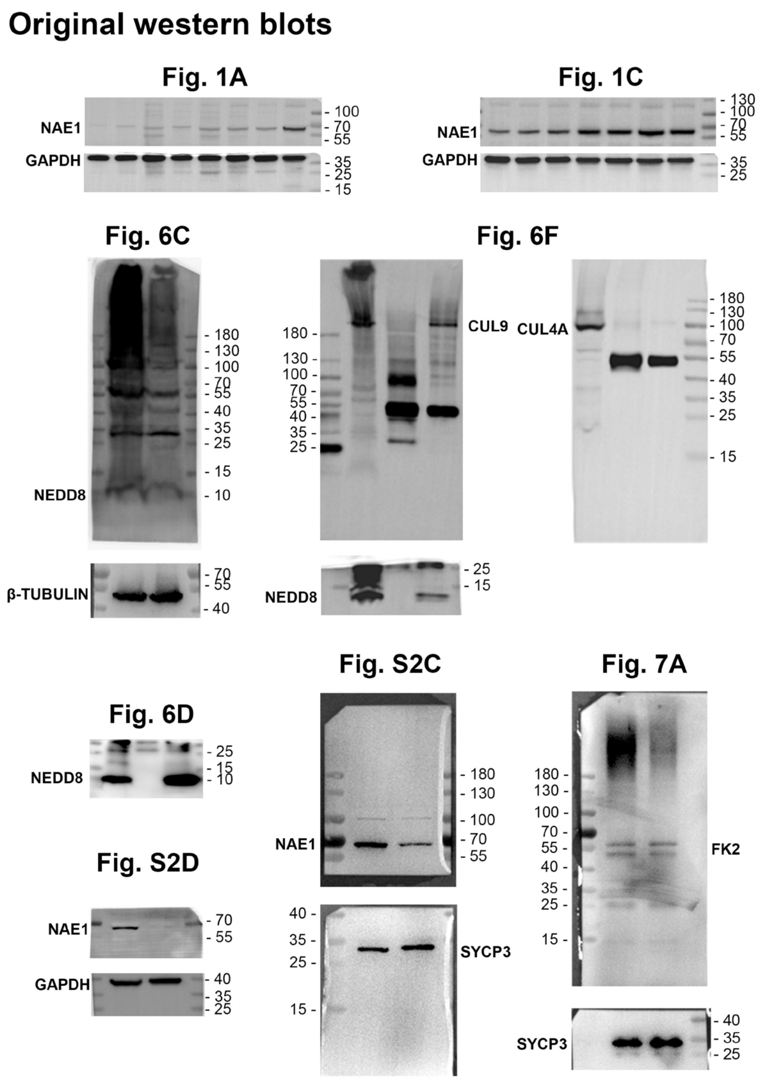

Figure S7. Original western blot images in this study.

Table S1. List of antibodies used in this study

Antibody	Manufacture (catalog number)	Source	Applications (working dilution)
SYCP3	Abcam (ab97672)	Mouse	IF (1:200) WB (1:1000)
SYCP3	Abcam (ab15093)	Rabbit	IF (1:200)
NAE1	Novus (NBP1-92162)	Rabbit	IF (1:200) WB (1:1000)
NEDD8	Abcam (ab81264)	Rabbit	IF (1:200) WB (1:1000)
γH2AX	Cell Signaling (#9718S)	Rabbit	IF (1:2000)
H1t	This paper	Guinea pig	IF (1:400)
SYCP1	Abcam (ab15090)	Rabbit	IF (1:200)
SIX6OSI	This paper	Rabbit	IF (1:200)
HORMAD1	Proteintech (13917-1-AP)	Rabbit	IF (1:300)
RPA2	Abcam (ab76420)	Rabbit	IF (1:100)
RAD51	Abcam (ab176458)	Rabbit	IF (1:100)
DMC1	Proteintech (13714-1-AP)	Rabbit	IF (1:100)
MLH3	Gifted by Mengcheng Luo	Rabbit	IF (1:100)
CDK2	Santa Cruz (sc-6248)	Rabbit	IF (1:50)
MSH4	Gifted by Qinghua Shi	Rabbit	IF (1:100)
TEX11	Gifted by Liangran Zhang	Rabbit	IF (1:200)
RNF212	Gifted by Mengcheng Luo	Rabbit	IF (1:100)
HEI10	Gifted by Hongbin Liu	Rat	IF (1:200)
CUL4A	Novus (NB100-2267)	Rabbit	WB (1:500)
CUL9	Thermofisher (A300-098A-T)	Rabbit	WB (1:500)
FK2	Millipore (#04-263)	Mouse	IF (1:200) WB (1:1000)
GAPDH	Trans (HC301)	Mouse	WB (1:1000)
β-tubulin	Trans (HC101)	Mouse	WB (1:3000)
Anti-mouse HRP	Trans (HS201)	Goat	WB (1:5000)
Anti-rabbit HRP	Trans (HS101)	Goat	WB (1:5000)
Anti-Rabbit IgG H&L (Alexa Fluor® 488)	Abcam (ab150065)	Goat	IF (1:200)
Anti-Mouse IgG H&L (Alexa Fluor® 594)	Abcam (ab150108)	Goat	IF (1:200)

Anti-Guinea pig IgG H&L (Alexa Fluor® 405)	Abcam (ab175678)	Goat	IF (1:200)
Anti- Rat IgG H&L (Alexa Fluor® 594)	Abcam (ab150152)	Donkey	IF (1:200)

Table S2. List of primers for genotyping and qPCR

Gene name	Forward primer	Reverse primer
Nae1 (genotyping)	CAGGTGTCTGCAGAACATTGGTTATAG	ACAGCTGATGTTAAGTCTCCTTGAAGGA
Nae1 (qPCR)	GCAACTCAGCTTCCTGAAAG	CTCTCAGTTCAGGAAATGGC
Stra8	ACTCCAAGCACTGGGCAGAA	(R1) GCCACCATAGCAGCATCAAA<br>(R2) CGTTTACGTCGCCGTCCAG
Dppa5a	TATTCCAGGTCCAGTCGCTG	TGAAGCATCCATTTAGCCCG
Ero1l	GCCTTGTCCTTTCTGGAATG	CAGAGACTCATCCACGGCTC
Ftl1	TTGATCGGGATGACGTGGCT	ATGGCCTCCTGGGTTTTACC
Sqstm1	GGAGCTGACAATGGCTATGT	CACACTGCACTTATAGCGAG
Actb	CCACACCTTCTACAATGAGC	CTCCGGAGTCCATCACAATG
